# High-Throughput Detection of Bacterial Community and Its Drug-Resistance Profiling From Local Reclaimed Wastewater Plants

**DOI:** 10.3389/fcimb.2019.00303

**Published:** 2019-10-04

**Authors:** Alya Limayem, Sarah Wasson, Mausam Mehta, Anaya Raj Pokhrel, Shrushti Patil, Minh Nguyen, Jing Chen, Bina Nayak

**Affiliations:** ^1^Graduate Program, Department of Pharmaceutical Sciences, College of Pharmacy, University of South Florida, Tampa, FL, United States; ^2^Division of Translational Medicine, Center for Education in Nanobioengineering, University of South Florida, Tampa, FL, United States; ^3^Morsani College of Medicine, University of South Florida, Tampa, FL, United States; ^4^College of Public Health, University of South Florida, Tampa, FL, United States; ^5^College of Arts and Sciences, University of South Florida, Tampa, FL, United States; ^6^Pinellas County Utilities, Water Quality Division, Largo, FL, United States

**Keywords:** treated wastewater, drug-resistance, pathogens, bacterial community structure, wastewater treatment

## Abstract

Treated wastewater from reclaimed facilities (WWTP) has become a reusable source for a variety of applications, such as agricultural irrigation. However, it is also a potential reservoir of clinically-relevant multidrug resistant (MDR) pathogens, including ESKAPE (*Enterococcus faecium* and *Streptococcus surrogates, Staphylococcus aureus, Klebsiella pneumoniae, Acinetobacter baumannii, Pseudomonas aeruginosa*, and *Enterobacter* species along with the emerging nosocomial *Escherichia* strains). This study was performed to decipher the bacterial community structure through Illumina high throughput 16S rRNA gene sequencing, and to determine the resistance profile using the Sensititre antimicrobial susceptibility test (AST) conforming to clinical lab standards (NCCLS). Out of 1747 bacterial strains detected from wastewater influent and effluent, *Pseudomonas* was the most predominant genus related to ESKAPE in influent, with sequence reads corresponding to 21.356%, followed by *Streptococcus* (6.445%), *Acinetobacter* (0.968%), *Enterococcus* (0.063%), *Klebsiella* (0.038%), *Escherichia* (0.028%) and *Staphylococcus* (0.004%). Despite the different treatment methods used, the effluent still revealed the presence of some *Pseudomonas* strains (0.066%), and a wide range of gram-positive cocci, including *Staphylococcus* (0.194%), *Streptococcus* (0.63%) and *Enterococcus* (0.037%), in addition to gram-negative *Acinetobacter* (0.736%), *Klebsiella* (0.1%), and *Escherichia* sub-species (0.811%). The AST results indicated that the strains *Escherichia* along with *Klebsiella* and *Acinetobacter*, isolated from the effluent, displayed resistance to 11 antibiotics, while *Pseudomonas* was resistant to 7 antibiotics, and *Streptococcus* along with *Staphylococcus* were resistant to 9 antibiotics. Results herein, proved the existence of some nosocomial MDR pathogens, known for ESKAPE, with potential drug resistance transfer to the non-pathogen microbes, requiring targeted remediation.

**Graphical Abstract:**
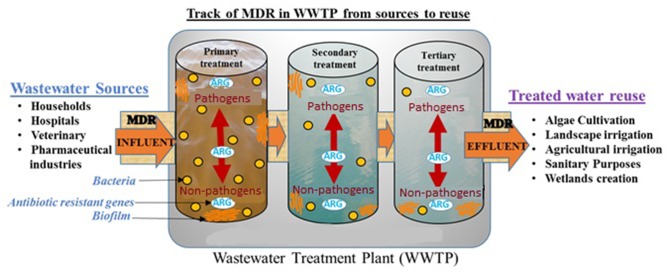
MDR pathogens spread from reclaimed wastewater into treated reusable water and surrounding environment such as agricultural irrigation.

## Introduction

Secondary or tertiary wastewater from WWTP has become a source of prime interest for various applications, encompassing primarily agricultural and landscape irrigation, as well as algae cultivation and sanitation (Sheehan, 1998; Jiménez and Asano, 2008; Woertz et al., 2009; Ferrell and Sarisky-Reed, 2010; Limayem et al., 2016). While the treated wastewater (TWW) generates considerable investment returns with minimal capital cost, it has become a tremendous reservoir for bacterial communities, including non-pathogens and pathogens of nosocomial origin, which carry and transfer antibiotic-resistant genes (Watkinson et al., 2007; Bouki et al., 2013; Limayem and Martin, 2014; Hong et al., 2018). The mixture of wastewater from different sources with hospital and pharmaceutical discharges is possibly one of the main causes for the exacerbation of multidrug resistance (MDR) in wastewater before it reaches the WWTP (Loraine and Pettigrove, 2006; Kim and Aga, 2007; Baquero et al., 2008; Rijal et al., 2009; Naidoo and Olaniran, 2013; Pruden et al., 2013; Everage et al., 2014; Berendonk et al., 2015; Walia et al., 2016). However, the untreated wastewater community structure varies from one region to another as it is extensively described by Shanks et al. (2013), suggesting localized screening to each specific region to generate an accurate conclusion on the community structure.

Fahrenfeld et al. (2013) have reported that despite wastewater treatment, there is re-growth of some pathogenic bacterial strains. They also demonstrated that even with recycled wastewater, some resistant genes would remain at the point of use (Pruden et al., 2006, 2013; McKinney et al., 2010; Ma et al., 2011; McKinney and Pruden, 2012; Fahrenfeld et al., 2013). This study herein, suggests that the recalcitrant flocs (biofilms) would protect MDR bacteria present in their core-center from disinfectants and available treatment could not transpierce the biofilm matrix to reach all the bacterial cells inside. Therefore, the surviving MDR bacteria could re-grow within different treatment stages and transfer their genetic material via horizontal gene transfer to other microorganisms, thus creating a selective pressure and a resilient breeding ground of resistant bacteria in WWTP (Arboleya et al., 2012; Jünemann et al., 2012; Bergeron et al., 2017).

To date, numerous studies have reported the prevalence of drug-resistant bacteria and genes in the environment (Pruden et al., 2006; Fahrenfeld et al., 2013; Garner et al., 2016; Hong et al., 2018; Yu et al., 2018). It is quite possible that reuse of water, along with natural disasters such as flooding or hurricanes, are major factors to the dissemination of MDR bacteria from the wastewater to the environment (Karam, 2006; Ferro et al., 2015; Garner et al., 2016, 2017; Yu et al., 2018). It would explain the unprecedented increasing toll on global death due to MDR infections, which causes 700,000 hospitalizations per annum and is expected to reach 10 million deaths by 2050 (O'Neill, 2016). There is, therefore, an exigency to identify and quantify MDR bacteria found in all treatment steps, to pinpoint the source and ensure traceability in an attempt to suppress the reoccurring cycle of resistance in WWTP.

Typically, the conventional 16S rRNA gene sequencing for bacterial identification is a low resolution technique that have prevented comprehensive characterization and requires analysis of a substantial number of samples for isolating and culturing individual species (Ma et al., 2015). Moreover, this process demands intensive labor with significant capital and time investments (Limayem et al., 2018). Using a rapid and less laborious advanced multiplexing system, namely the high throughput Illumina 16S rRNA sequencing of the entire cultivable and uncultivable bacterial community can simultaneously be deciphered in one single sample (Limayem et al., 2018). The Illumina is an advanced, cost-effective, and less labor intensive tool with the capability to take a snapshot of extensive data under changing factors such as temperature. It immobilizes random DNA surface fragments followed by PCR amplification, which results in identical DNA fragment clusters. With a read length of only 150 bp, this system is able to sequence clusters from both ends of the fragments, and also offers a faster run time compared to other systems (Torsten et al., 2012).

For this purpose, influent and effluent wastewater samples were collected from a local WWTP to elucidate the bacterial community profiling at the first point of comparison and control. The bacterial profiling was determined in one single high throughput, multiplexing identification with the awareness that even with the same conditions (i.e., temperature, load, time…), the community structure can vary from one investigation to another. The specific focus of this research study was to confirm the presence of pathogens of nosocomial origin carrying multiple drug resistance via advanced high-throughput Illumina 16S rRNA gene sequencing and to screen for their antimicrobial susceptibility profile via the Vizion Sensititre system CMV3AGPF including 16 antibiotic agents. This panel has the most broad spectrum of antibiotics for screening both gram-positive and gram-negative bacteria. The identification and quantification of some pathogens, carrying resistant genes, should warrant the unmet need for a targeted nano-treatment to eradicate the resistance cycle from the sources to WWTP and downstream applications.

## Materials and Methods

The influent and effluent samples were kindly donated to our lab by a local WWTP in Florida. Total of 1.5 L of influent and 6 L of effluent wastewater were collected into sterile plastic containers. The water samples were transported to the laboratory on ice pack within 2 h of collection and sent for sequencing immediately. Any remaining samples were stored at 4°C until next day for bacterial culture. The plant had two reclamation facilities and provided us with representative sample batches including primarily wastewaters from residential and hospital origin. The sampled effluent, which is used for irrigation purposes, received both secondary treatment, involving clarifiers and activated sludge treatment using oxic/anoxic tanks, and tertiary treatment, using deep bed denitrification filters and disinfection trains.

### DNA Extraction, High-Throughput 16S rRNA Sequencing, and Statistical Analysis

The Genomic DNA was prepared from the wastewater samples using standard protocol (Venter et al., 2004). The TruSeq Genomic DNA HT Sample Prep Kit (Illumina, cat. No. FC-102-1001) was used for isolation and purification of bacterial DNA. Sample preparation was optimized so that the amount of samples as less as tens of nanogram would be adequate for sequencing single-end library (Torsten et al., 2012). From the prepared genomic DNA, 5 μg/ml of DNA with OD260/280 ratio of 1.8–2 was used for library preparation. The DNA libraries were prepared by adding adapter sequences that correspond to the two surface-bound amplification primers on the flow cells used in the cluster generation, onto the ends of DNA fragments. The prepared libraries were sequenced on the Illumina Miseq, version 1.0.1.0 using a 600 cycle V3 standard flow cell producing ~100,000 paired-end 2 × 300 base reads (Omega Bioservices, Norcross, GA). The sequence reads passing quality filtering were used for classification at each taxonomic level. The 16S rRNA gene sequencing results were analyzed via Illumina's BaseSpace 16S rRNA application module, the Illumina-curated version of the May 2013 Greengenes taxonomic database.

An optimized version of the Ribosomal Database Project Naïve Bayes taxonomic classification algorithm was used to obtain rapid and accurate classification of sequence reads. For each sample, the raw reads were filtered based on sequencing quality using Trimmomatic v0.30, involving removal of primer and adaptor sequence and truncation of sequence reads with both pair end quality <25 nucleotides. QIIME pipeline was used to perform the ITS analysis. Sequences were clustered into operational taxonomic units (OTU) at a 97% similarity cutoff and the relative abundance was calculated for each sample. All sequences were classified using a native Bayesian classifier (Wang et al., 2007) trained against the Ribosomal Database Project training set. The OTU sequences were aligned to the Silva database to create a phylogenetic tree and an OTU table was prepared, representing the abundance of each OTU in each microbial sample (Yilmaz et al., 2014). The alpha diversity (within community diversity) refraction curves (graphs of diversity vs. sequencing depth; Quast et al., 2013) and beta diversity analysis was performed for each of the microbial communities.

Two different methods were implemented to isolate multi-drug resistant bacteria of nosocomial origin from WWTP samples such as *Pseudomonas* and *Streptococcus*. In the first method, the wastewater samples were spun down in the centrifuge at 3,396 G for 10 min to concentrate the samples up to 300 times. The concentrated samples were diluted by a factor of 3x and 30x in separate tubes with tryptic soy broth (TSB). The separate tryptic soy agar (TSA) plates were streaked with each dilution and the plates were incubated at 37°C for 24 h. Another method of isolation mixed 500 μl of 4x concentrated TSB media in 1.5 mL of wastewater samples in eight tubes followed by incubation at 37°C for 72 h. After incubation, three TSA plates were streaked directly from the broth cultures and incubated at 37°C for 24 h.

### Bacterial Colony Isolation

The colonies that appeared on the TSA plates were streaked for isolation on a number of selective and differential media [i.e., MacConkey, Mannitol Salt, Xylose Lysine Deoxycholate, Salmonella-Shigella, Eosin Methylene Blue, De Man Rogosa Sharpe (MRS)], in accordance with the National Committee for Clinical Laboratory Standards definitions (NCCLS). The plates were incubated at 37°C for 24 h. The colors and morphologies of the colonies were noted from the selective plates. Individual colonies from each of the selective plate were cultured in 2 mL TSB and incubated at 37°C for 24 h. A loopful of bacteria from each pure culture was then streaked onto its respective TSA plate for the final steps of isolation and for subsequent use with Antibiotic Sensitivity Test (AST). Plates were incubated at 37°C for 24 h.

### Antimicrobial Susceptibility Testing

The AST was determined through the Clinical and Laboratory Sciences Institute (CLSI) certified Vizion Sensititre (Thermo Fisher Scientific, Pittsburgh, PA.) using the Sensititre NARMS Plate (cat#: CMV3AGPF). The colonies on the TSA plate streaked from a pure culture were collected from the agar plate and mixed into a sterile tube containing 5 mL deionized water. The suspension was vortexed for 15 s and turbidity was adjusted to 0.5 McFarland standard using calibrated Sensititre Nephelometer (Trek Diagnostic Systems). The 10 μl of the suspension was transferred to 11 mL of Tryptic soy broth (TSB) to prepare the culture for AST. Using the Sensititre multichannel pipette, a 50 μL of the culture was loaded into each well of CMV3AGPF panel (Trek Diagnostic Systems) plates. This procedure was repeated for each of the bacterial isolate, for a total of 10 isolates. Plates were sealed and incubated at 37°C for 24 h followed by screening for antibiotic resistance using the Vizion Sensititre System and SWIN software. The Vizion Sensititre manual for reading of the panel following the basic guidelines was used.

### Characterization of MDR Isolates

AST test was performed for each morphologically unique isolate. The CMV3AGPF panel contained various concentrations of sixteen different antibiotics (i.e., Chloramphenicol, Penicillin, Ciprofloxacin, Daptomycin, Linezolid, Erythromycin, Gentamicin, Kanamycin, Nitrofurantoin, Tigecycline, Tylosin (Tartrate/ Base), Quinupristin/dalfopristin, Lincomycin, Streptomycin, Tetracycline, and Vancomycin). The genomic DNA from antibiotic resistant isolates were used for 16S rRNA sequencing and analysis. The gene amplification was carried out using 8F and 1492R universal primers (8F: 5′ AGAGTTTGATCCTGGCTCAG 3′ and 1492R: 5′ ACCTTGTTACGACTT 3′). The PCR conditions used for amplification were as stated: an initial denaturation of 95°C for 3 min followed by 25 cycles of denaturation at 95°C for 30 s, annealing at 55°C for 30 s, extension at 72°C for 30 s and a final extension at 72°C for 5 min. The sequenced 16S rRNA gene was subjected to taxonomic classification using resources from the Ribosomal Database Project (Cole et al., 2009). The taxonomy data was further verified by comparison with EzBioCloud taxonomically united 16S rRNA database (Yoon et al., 2017) and NCBI 16S ribosomal RNA sequence database.

## Results

### High-throughput Profiling of Bacterial Diversity on Influent and Effluent Wastewater

**Figure 1:**
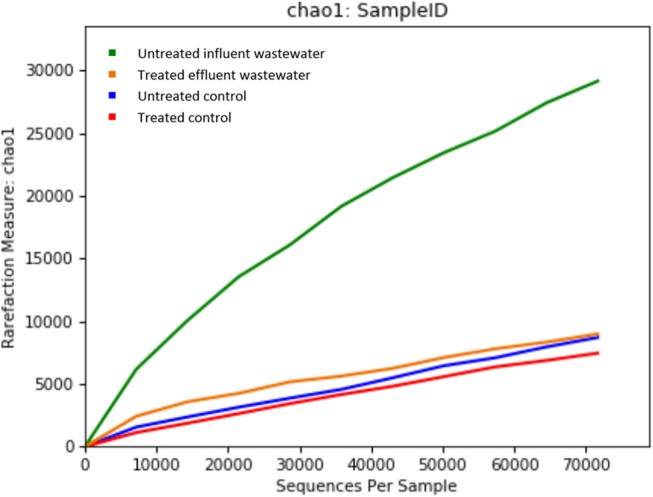
Alpha rarefaction curves calculated for **(A)** chao1 index demonstrating higher diversity of isolates in untreated influent and **(B)** observed OTU diversity between untreated influent and treated effluent samples.

Metagenomics is a powerful tool, which provides access to the functional genetic composition of microbial communities and development of novel hypotheses on microbial function (Torsten et al., 2012). All of the reads from both effluent (150,341 reads) and influent (195,967 reads) WW samples passed the quality control filters. The sequence data was grouped based on OTU for effluent and influent samples. The refraction curve was plotted based on comparison of different numbers of species and sequence from each sample contained in OTU. The curve shows sufficient number of sequence reads for optimum species diversity for both effluent and influent samples (Figure 1).

**Figure 2:**
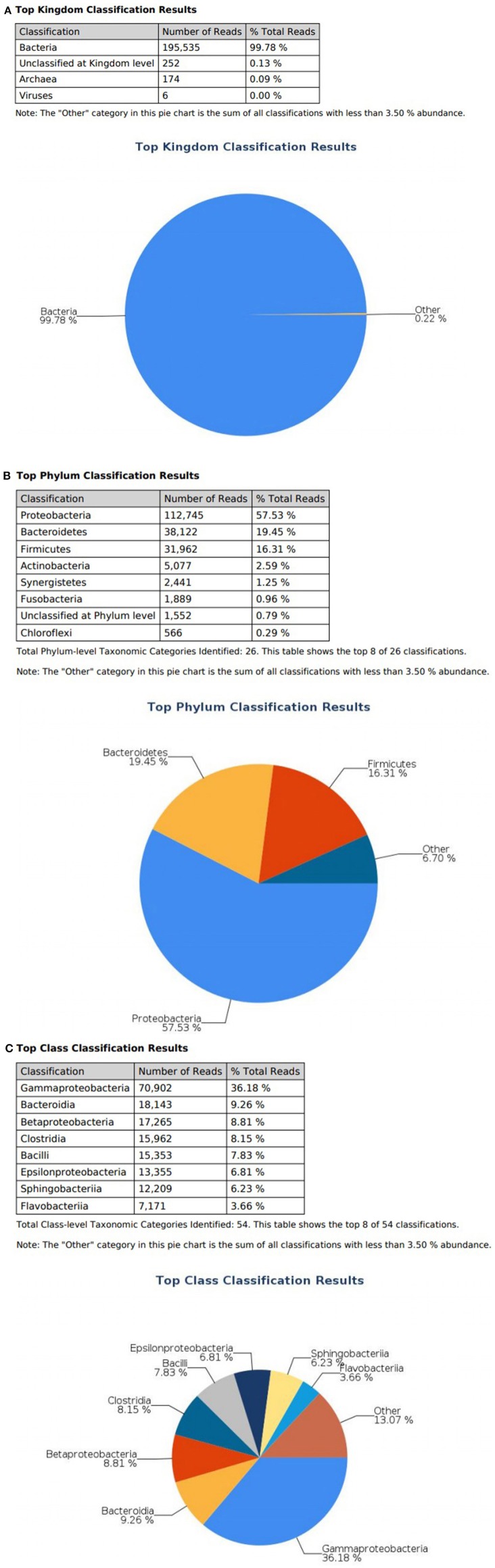
illumina® 16S Metagenomic report on influent wastewater; classification at the level of: **(A)** Kingdom **(B)** Phylum **(C)** Class.

**Figure 3:**
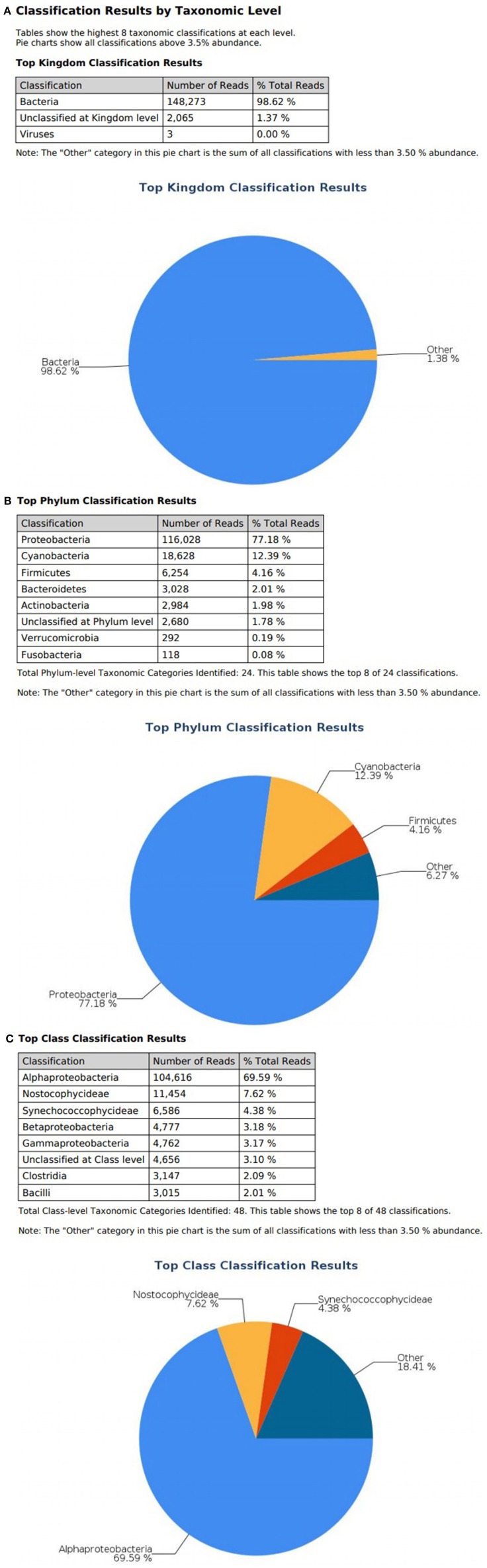
illumina® 16S Metagenomic report on effluent wastewater; classification at the level of: **(A)** Kingdom **(B)** Phylum **(C)** Class.

At the domain level, 99.78 and 98.62% of sequence reads belonged to bacteria while 0.13% and 1.37% of reads were unclassified in influent and effluent samples, respectively (Figures 2A, 3A; Tables 1, 2). The focus is on the phylum of Proteobacteria and Firmicutes in which ESKAPE species belong to. Proteobacteria consist of 57.53% (112,745) of the readings in influent wastewater while Firmicutes only yielded 16.31% (31,962) (Figure 2B). The effluent wastewater yielded increased readings for Proteobacteria at 77.18% (116,028) but decreased readings for Firmicutes at 4.16% (6,254) (Figure 3B).

**Table 1:** Classification statistics of each taxonomic level.

**Taxonomic Level**	**Total reads (untreated influent)**	**Percentage total reads**	**Total reads (treated effluent)**	**Percentage total reads**
Domain	195,715	99.87%	148,276	98.63%
Phylum	194,415	99.21%	147,661	98.22%
Class	192,955	98.46%	145,685	96.90%
Order	191,962	97.96%	145,406	96.72%
Family	189,908	96.91%	144,880	96.37%
Genus	184,240	94.02%	143,449	95.45%
Species	119,295	60.88%	126,843	84.37%

**Table 2:** Top classifications in untreated influent and treated effluent.

**Taxonomic Level**	**Top Classification Identity**	**Percentage total reads (influent)**	**Percentage total reads (effluent)**
Domain	Bacteria	99.78%	98.62%
Phylum	Proteobacteria	57.53%	77.18%
Class	Gammaproteobacteria	36.18%	3.17%
	Alphaproteobacteria	1.90%	69.59%
Order	Pseudomonadales	27.60%	2.73%
	Rhodobacterales	0.60%	60.29%
Family	Pseudomonadaceae	25.39%	1.80%
	Rhodobacteraceae	0.60%	59.69%
Genus	*Paracoccus*	38.845%	1.535%
	*Pseudomonas*	21.356%	0.066%
Species	Unclassified	39.12%	15.63%
	*Paracoccus marcusii*	38.684%	0.353%

On the class level, Gammaproteobacteria and Bacilli contain ESKAPE strains. Gammaproteobacteria consist 36.18% (70,902) of influent wastewater while Bacilli make up 7.83% (15,353) (Figure 2C). In effluent wastewater, Gammaproteobacteria only consist 3.17% (4,762). Interestingly, there is a major component of Alphaproteobacteria at 69.59% (104,616) and Betaproteobacteria at 3.18% (4,777) in effluent wastewater that made up an insignificant amount in influent wastewater. There is also a decrease in Bacilli which showed in 2.01% (3,015) of the readings (Figure 3C).

At the genus level there are significant readings of genera of interest which encompass ESKAPE species. In influent wastewater, *Pseudomonas* had 21.356% (41,850) of the total readings, while *Acinetobacter* had 1,897 total readings (0.968%) and *Streptococcus* had 12,630 readings (6.445%). Furthermore, *Enterococcus* had 124 total readings (0.063%) while *Escherichia* and *Staphylococcus* on the other hand had 54 (0.028%) and 8 (0.004%), respectively (Tables 3–5). In effluent wastewater, while the *Pseudomonas, Streptococcus* and *Acinteobacter* readings decreased to 99 (0.066%), 947 (0.63%) and 1107 (0.736%), respectively, as well as *Enterococcus* to 55 (0.037%), the readings for *Klebsiella, Escherichia* and *Staphylococcus* increased to 151 (0.1%), 1219 (0.811%), and 291 (0.194%), respectively (Tables 3–5).

**Table 3:** Percentage read distribution from phylum level to genera *Pseudomonas* and *Acinetobacter*.

**Classification**	**%reads on influent**	**%reads on effluent**
Phylum Proteobacteria	57.53%	77.18%
Class Gammaproteobacteria	36.18%	3.17%
Order Pseudomonadales	27.60%	2.73%
Family		
1) Pseudomonadaceae 2)Moraxellaceae	25.39% 1.044%	1.80% 0.742%
Genus		
1)*Pseudomonas*	21.356%	0.066%
2)*Acinetobacter*	0.968%	0.736%

**Table 4:** Percentage read distribution from phylum level to genera *Escherichia* and *Klebsiella*.

**Classification**	**%reads on influent**	**%reads on effluent**
Phylum Proteobacteria	57.53%	77.18
Class Gammaproteobacteria	36.18%	3.17%
Order Enterobacteriales	3.60%	1.87%
Family Enterobacteriaceae	2.30%	1.90%
Genus		
1)*Escherichia* 2) *Klebsiella*	0.028% 0.038%	0.811% 0.1%

**Table 5:** Percentage read distribution from phylum level to genera *Streptococcus, Staphylococcus*, and *Enterococcus*.

**Classification**	**%reads on influent**	**%reads on effluent**
Phylum Firmicutes	16.31%	4.16%
Class Bacilli	7.83%	2.01%
Order Lactobacillales	7.57%	1.90%
Family		
1) Streptococcaceae 2) Staphylococcaceae 3)Enterococcaceae	6.85% 0.005% 0.070%	1.70% 0.194% 0.041%
Genus		
1) *Streptococcus* 2) *Staphylococcus* 3)*Enterococcus*	6.445% 0.004% 0.063%	0.63% 0.194% 0.037%

The following is the reading for individual ESKAPE species. *Enterococcus faecium* had 1 reading in influent wastewater (0.001%) and none in effluent wastewater. *Staphylococcus aureus* had 3 readings in influent wastewater (0.002%) and 207 readings in effluent wastewater (0.138%). *Klebsiella pneumonia* had 9 readings for influent (0.005%) and 27 readings for effluent wastewater (0.018%). *Acinetobacter baumannii* had no readings in influent wastewater and 4 readings (0.003%) in effluent wastewater. *Pseudomonas* aeruginosa had 2 readings in influent wastewater (0.001%) 7 readings in effluent wastewater (0.005%). Table 6 shows the taxonomical list of species of interest, including the detected ESKAPE pathogen counts, while Supplementary Figure 1 provides the complete list of the 1747 identified species.

**Table 6:** Taxonomy list of MDR species identified in both influent and effluent wastewater isolates and potential ESKAPE species.

**Phylum**	**Class**	**Genus**	**Species**	**Potential ESKAPE with number of readings (influent/effluent)**
Proteobacteria	Gammaproteobacteria	*Klebsiella* (0.038% influent, 0.1% effluent)	*Klebsiella* variicola	*Klebsiella* pneumonia (9/27)
			*Klebsiella* oxytoca	
			***Klebsiella*** **pneumoniae**	
			*Klebsiella* granulomatis	
		*Pseudomonas* (21.356% influent, 0.066% effluent)	*Pseudomonas* ludensis	*Pseudomonas* aeruginosa (2/7)
			*Pseudomonas* plecoglossicida	
			*Pseudomonas* fragi	
			*Pseudomonas* marginalis	
			*Pseudomonas* tremae	
			*Pseudomonas* veronii	
			*Pseudomonas* brenneri	
			*Pseudomonas* cremoricolorata	
			*Pseudomonas* mandelii	
			*Pseudomonas* proteolytica	
			*Pseudomonas* benzenivorans	
			*Pseudomonas* azotoformans	
			*Pseudomonas* fluorescens	
			*Pseudomonas* pseudoalcaligenes	
			*Pseudomonas* vancouverensis	
			*Pseudomonas* mosselii	
			*Pseudomonas* moraviensis	
			*Pseudomonas* savastanoi	
			*Pseudomonas* putida	
			*Pseudomonas* umsongensis	
			*Pseudomonas* coronafaciens	
			*Pseudomonas* oryzihabitans	
			*Pseudomonas* corrugata	
			*Pseudomonas* syncyanea	
			*Pseudomonas* alcaligenes	
			*Pseudomonas* parafulva	
			*Pseudomonas* anguilliseptica	
			*Pseudomonas* panipatensis	
			*Pseudomonas* guinea	
			*Pseudomonas* clemancea	
			*Pseudomonas* teessidea	
			*Pseudomonas* poae	
			*Pseudomonas* metavorans	
			*Pseudomonas* syringae	
			*Pseudomonas* viridiflava	
			*Pseudomonas* meliae	
			*Pseudomonas* migulae	
			*Pseudomonas* mendocina	
			*Pseudomonas* stutzeri	
			*Pseudomonas* lini	
			*Pseudomonas* xylanivorans	
			*Pseudomonas* tropicalis	
			*Pseudomonas* entomophila	
			*Pseudomonas* collierea	
			*Pseudomonas* rhodesiae	
			*Pseudomonas* brassicacearum	
			***Pseudomonas*** **aeruginosa**	
			*Pseudomonas* gessardii	
			*Pseudomonas* agarici	
			*Pseudomonas* cinnamophila	
			*Pseudomonas* lurida	
			*Pseudomonas* alkylphenolia	
			*Pseudomonas* resinovorans	
			*Pseudomonas* otitidis	
			*Pseudomonas* jessenii	
			*Pseudomonas* trivialis	
			*Pseudomonas* cichorii	
			*Pseudomonas* tolaasii	
			*Pseudomonas* fuscovaginae	
			*Pseudomonas* reinekei	
			*Pseudomonas* mucidolens	
			*Pseudomonas* xanthomarina	
			*Pseudomonas* chloritidismutans	
			*Pseudomonas* psychrophila	
			*Pseudomonas* panacis	
			*Pseudomonas* amygdali	
			*Pseudomonas* taiwanensis	
			*Pseudomonas* gingeri	
			*Pseudomonas* jinjuensis	
			*Pseudomonas* koreensis	
			*Pseudomonas* pavonaceae	
			*Pseudomonas* mediterranea	
			*Pseudomonas* thermotolerans	
			*Pseudomonas* japonica	
			*Pseudomonas* borealis	
			*Pseudomonas* fulva	
			*Pseudomonas* moorei	
			*Pseudomonas* caricapapayae	
		*Escherichia* (0.028% influent, 0.811% effluent)	*Escherichia* albertii	*Escherichia* coli (4/33)
			***Escherichia*** **coli**	
		*Shigella*	N/A	N/A
		*Salmonella*	*Salmonella enterica*	N/A
		*Acinetobacter* (0.968% influent, 0.736% effluent)	*Acinetobacter* guillouiae	*Acinetobacter* baumannii (0/4)
			*Acinetobacter* johnsonii	
			*Acinetobacter* seohaensis	
			*Acinetobacter* gerneri	
			*Acinetobacter* tjernbergiae	
			*Acinetobacter* psychrotolerans	
			*Acinetobacter* antiviralis	
			*Acinetobacter* bouvetii	
			*Acinetobacter* indicus	
			*Acinetobacter* schindleri	
			*Acinetobacter* hemolyticus	
			*Acinetobacter* beijerinckii	
			*Acinetobacter* marinus	
			*Acinetobacter* glacincola	
			*Acinetobacter* lwoffii	
			*Acinetobacter* xiamenensis	
			*Acinetobacter* junii	
			***Acinetobacter*** **baumannii**	
			*Acinetobacter* rhizosphaerae	
			*Acinetobacter* ursingii	
			*Acinetobacter* radioresistens	
			*Acinetobacter* tabacinasalis	
			*Acinetobacter* oleivorans	
			*Acinetobacter* gyllenbergi	
			*Acinetobacter* baylyi	
	Alpha proteobacteria	*Sphingobium*	*Sphingobium* yanoikuyae	N/A
			*Sphingobium* amiense	
			*Sphingobium* olei	
			*Sphingobium* faniae	
			*Sphingobium* ummariense	
			*Sphingobium* abikonense	
			*Sphingobium* rhizovicinum	
		*Sphingomonas*	*Sphingomonas* oligophenolica	N/A
			*Sphingomonas* panni	
			*Sphingomonas* insulae	
			*Sphingomonas* dokdonensis	
			*Sphingomonas* ginsenosidimutans	
			*Sphingomonas* melonis	
			*Sphingomonas* azotifigens	
			*Sphingomonas* hunanensis	
			*Sphingomonas* abaci	
			*Sphingomonas* wittichii	
			*Sphingomonas* sanxanigenens	
			*Sphingomonas* elodea	
			*Sphingomonas* hankookensis	
			*Sphingomonas* roseiflava	
			*Sphingomonas* japonica	
			*Sphingomonas* asaccharolytica	
			*Sphingomonas* mali	
			*Sphingomonas* yunnanensis	
			*Sphingomonas* soli	
			*Sphingomonas* yabuuchiae	
			*Sphingomonas* haloaromaticamans	
Firmicutes	**Bacilli**	*Staphylococcus* (0.004% influent, 0.194% effluent)	***Staphylococcus aureus***	*Staphylococcus aureus* (3/207)
			*Staphylococcus* lugdunensis	
			*Staphylococcus* caprae	
			*Staphylococcus* hominis	
			*Staphylococcus* epidermidis	
			*Staphylococcus* pseudolugdunensis	
			*Staphylococcus* capitis	
			*Staphylococcus* haemolyticus	
			*Staphylococcus warneri*	
			*Staphylococcus* gallinarum	
			*Staphylococcus* pasteuri	
			*Staphylococcus* cohnii	
			*Staphylococcus* auricularis	
		*Streptococcus*	*Streptococcus* minor	N/A
			*Streptococcus* bovis	
			*Streptococcus* equinus	
			*Streptococcus* vestibularis	
			*Streptococcus* tigurinus	
			*Streptococcus* oralis	
			*Streptococcus* gordonii	
			*Streptococcus* pseudopneumoniae	
			*Streptococcus* infantis	
			*Streptococcus* parasanguinis	
			*Streptococcus* thermophilus	
			*Streptococcus* fryi	
			*Streptococcus* cristatus	
			*Streptococcus* australis	
			*Streptococcus* sanguinis	
			*Streptococcus* gallinaceus	
			*Streptococcus* infantarius	
			*Streptococcus* orisratii	
			*Streptococcus* milleri	
			*Streptococcus* ferus	
			*Streptococcus* peroris	
			*Streptococcus* alactolyticus	
			*Streptococcus* lactarius	
			*Streptococcus* mutans	
			*Streptococcus* castoreus	
			*Streptococcus* ursoris	
			*Streptococcus* anginosus	
			*Streptococcus* canis	
			*Streptococcus* phocae	
			*Streptococcus* dentirousetti	
			*Streptococcus* labedae	
			*Streptococcus* roseogilvus	
			*Streptococcus* intermedius	
			*Streptococcus* plurextorum	
			*Streptococcus* marimammalium	
			*Streptococcus* troglodytae	
			*Streptococcus* oligofermentans	
			*Streptococcus* dentapri	
			*Streptococcus* agalactiae	
Actinobacteria	Actinobacteria	***Micrococcus***	*Micrococcus yunnanensis*	N/A
			*Micrococcus luteus*	
Cyanobacteria	Cyanophyceae	***Calothrix***	*Calothrx* parietina	N/A
		***Leptolyngbya***	*Leptolyngbya* laminosa	N/A
			*Leptolyngbya antarctica*	

*The bold values indicate the relevant ESKAPE species found in the taxonomic reading of the influent and effluent wastewater*.

### Characterization of MDR Isolates

A series of serial dilutions, plating and resuspension was performed to collect the homogenous colonies of pure culture on individual selective media. The most dilute inoculum yielded colonies with the lowest CFU counts, and were small and well-separated (Janssen et al., 2002). The concentration of antibiotic agents that inhibited the growth of bacteria were obtained from the Sensititre plate reading (Table 7).

**Table 7:** Inhibitory concentration range of antibiotic agents against potential ESKAPE isolates.

**Antibiotic agents**	**Cocci species (*Streptococcus*/ *Staphylococcus)* MIC range (μg/ml)**	***Pseudomonas* species MIC range (μg/ml)**	***Escherichia* species MIC range (μg/ml)**	***Klebsiella* species MIC range (μg/ml)**	***Acinetobacter* species MIC range (μg/ml)**
Chloramphenicol	8–16	2–4	>32	>32	>32
Ciprofloxacin	0.5–1	0.5–1	>4	>4	>4
Daptomycin	8–16	8–16	>16	>16	>16
Erythromycin	<0.25	1–2	>8	>8	>8
Gentamycin	<128	<128	<128	<128	<128
Kanamycin	512–1024	<128	<128	<128	<128
Streptomycin	<1	<1	<1	<1	<1
Lincomycin	4–8	4–8	>8	>8	>8
Linezolid	4–8	4–8	>8	>8	>8
Nitrofurantoin	32–64	32–64	>64	>64	>64
Penicillin	8–16	0.5–1	>16	>16	>16
Quinupristin/Dalfopristin	16–32	4–8	>32	>32	>32
Tertacycline	2–4	<1	4–8	4–8	4–8
Tigecycline	0.12–0.25	0.12–0.25	0.25–0.5	0.25–0.5	0.25–0.5
Tylosin tartrate	16–32	8–16	>32	>32	>32
Vancomycin	16–32	8–16	>32	>32	>32

The isolates from wastewater samples were subjected to resequencing of 16S rRNA gene amplicon for identification of the strain to the genus level. The analysis of sequence result confirmed the identity of six out of ten isolates to be related to ESKAPE, including *Streptococcus* spp., *Staphylococcus* spp., *Pseudomonas* spp., *Klebsiella* spp., *Acinetobacter* spp., and *Escherichia* spp. The MIC values of the antibiotics tested after 24 h incubation at 37°C are shown in Table 7. Both *Streptococcus* and *Staphylococcus* were susceptible to Erythromycin, Gentamycin, and Streptomycin with MIC values of <0.25, <128, and <1 μg/mL, respectively. The *Streptococcus* and *Staphylococcus* strains displayed medium tolerance to Chloramphenicol, Ciprofloxacin, Tetracycline and Tigecycline exhibiting the MIC values 8 to 16, 0.5 to 1, 2 to 4, and 0.12 to 0.25 μg/mL, respectively. However, the strains displayed resistance against the following antibiotics with MIC values: Daptomycin (8–16 μg/mL), Kanamycin (512–1,024 μg/mL), Lincomycin (4–8 μg/mL), Linezolid (4–8 μg/mL), Nitrofurantoin (32–64 μg/mL), Penicillin (8–16 μg/mL), Quinupristin/dalfopristin (16–32 μg/mL), Tylosin tartrate (16–32 μg/mL), and Vancomycin (16–32 μg/mL) (Supplementary Figure 2). *Pseudomonas* spp. was susceptible to the following antibiotics with respective MIC values: Chloramphenicol (2–4 μg/mL), Gentamycin (<128 μg/mL), Kanamycin (<128 μg/mL), and Streptomycin (<1 μg/mL). The *Pseudomonas* strain displayed moderate to complete resistance against following antibiotics with MIC values: Ciprofloxacin (0.5–1 μg/mL), Erythromycin (1–2 μg/mL), Daptomycin (8–16 μg/mL), Lincomycin (4–8 μg/mL), Linezolid (4–8 μg/mL), Nitrofurantoin (32–64 μg/mL), and Tigecycline (0.12–0.25 μg/mL) (Supplementary Figure 3). *Escherichia* spp as well as *Klebsiella* spp. and *Acinetobacter* spp. were isolated from effluent wastewater and were found to be the most resistant strains, with resistances to chloramphenicol, ciprofloxacin, daptomycin, erythromycin, lincomycin, linezolid, nitrofurantoin, penicillin, quinupristin/dalfopristin, tylosin tartrate, and vancomycin. The MIC values for the antibiotics tested were: Chloramphenicol (>32 μg/mL), Ciprofloxacin (>4 μg/mL), Daptomycin (>16 μg/mL), Erythromycin (>8 μg/mL), Gentamycin (<128 μg/mL), Kanamycin (<128 μg/mL), Streptomycin (<1 μg/mL), Lincomycin (>8 μg/mL), Linezolid (>8 μg/mL), Nitrofurantoin (>64 μg/mL), Penicillin (>16 μg/mL), Quinupristin /Dalfopristin (>32 μg/mL), Tertacycline (4–8 μg/mL), Tigecycline (0.25–0.5 μg/mL), Tylosin tartrate (>32 μg/mL), Vancomycin (>32 μg/mL) (Supplementary Figure 4).

## Discussion

Illumina high throughput 16S rRNA gene sequencing was used to elucidate the microbial composition and structure of the WWTP by filtering out low quality sequences, and ~150,341 reads and 195,967 reads were obtained from effluent and influent wastewater, respectively (Ma et al., 2015). Based on the results (as depicted in Table 1), the percentage of total reads decreased as the taxonomy went from domain level to species level. The resistant bacterial species were abundant in influent wastewater as compared to effluent sample however, the resistant isolates in effluent was focused on this study as it relates to public health (Table 7). Higher bacterial species diversity was observed in influent wastewater as compared to effluent sample with 1,324 bacterial species in influent wastewater compared to 848 species in effluent sample. The resequencing of 16S rRNA genes led to the identification of MDR isolates as subspecies of *Pseudomonas, Streptococcus, Staphylococcus, Klebsiella, Acinetobacter*, and *Escherichia*. The abundance of these subspecies in effluent from WWTP is in agreement with previous studies on wastewater samples from municipal wastewater plants (Yu and Zhang, 2012; Cydzik-Kwiatkowska and Zielinska, 2016). On EMB agar, the Gram negative colonies showing dark spots and a metallic green sheen were identified as *Escherichia* subspecies (Antony et al., 2016) while the clear Gram negative colonies on EMB agar were found to be *Pseudomonas* subspecies (Chiang et al., 2010) upon further analysis with 16S rRNA sequencing. Similarly, the Gram-positive isolates on TSA plates were identified as *Streptococcus* subspecies, while large pink colonies were identified as *Klebsiella* spp on MacConkey agar (Bagley and Seidler, 1978), and large yellow colonies were identified to be *Staphylococcus* spp. on MSA media (Kateete et al., 2010), with confirmation from 16S rRNA sequencing. Finally, *Acinetobacter* colonies were identified as lightly-colored and small colonies grown on 5% sheep blood agar (Bouvet and Grimont, 1986) upon further analysis with 16S rRNA sequencing.

Considering the richness of the WW microbial community, OTU fell within variability of control community sequence analysis. This suggested sufficiently prudent quality of MiniSeq 16S rRNA gene data (Edgar, 2013). Moreover low quality sequences were discarded during processing of 16S rRNA sequencing data, by maintaining the maximum expected error threshold (E-max; Pichler et al., 2018). The total reads passing standard quality controls were classified taxonomically. This led to the classification of more than 90% of sequence reads up to genus level (Table 2). However, only 60.88% of sequence reads from influent wastewater sample and 84.37% sequence reads from effluent sample were identified at species level, with 39.12% being unclassified in the influent sample and 15.63% being unclassified in the effluent sample (Table 2). This result shows greater diversity of bacterial species in WW samples than the ones that have been identified so far.

*Pseudomonas* belongs to phylum Proteobacteria, class Gammaproteobacteria, order Pseudomonadales, and family Pseudomonadaceae. The phylum Proteobacteria was most abundant with 57.53% of sequence reads on influent wastewater and 77.18% of sequence reads on effluent sample. The class Gammaproteobacteria was highly abundant on influent wastewater with 36.18% of sequence reads while sequence reads corresponding to the Gammaproteobacteria on effluent sample dropped to 3.17%. At the order level, Pseudomonadales was the most abundant in influent wastewater sample with 27.60% of sequence reads while the percentage read reduced to 2.73% in effluent sample. Pseudomonadaceae was the most prominent family with 25.39% corresponding sequence reads in influent wastewater sample, but reduced to 1.80% in effluent sample. Pseudomonas was the second most prominent genus on influent wastewater samples with 21.356% of sequence reads while the sequence reading was 0.066% in effluent sample. The *Pseudomonas* species were prevalent in the influent wastewater sample however the abundance was largely reduced in the effluent sample (Table 3). This result was in accordance with our previous study with municipal WW (Limayem et al., 2018). Genus *Acinetobacter* shares same phylum, class and order as *Pseudomonas* spp. (phylum Proteobacteria, class Gammaproteobacteria, order Pseudomonadales), with a different family (i.e., Moraxellaceae), and yielded percentage reads of 0.968 and 0.736% in influent and effluent samples, respectively.

*Escherichia* and *Klebsiella* subspecies are classified under phylum Proteobacteria, class Gammaproteobacteria, order Enterobacteriales, and family Enterobacteriaceae. The phylum Proteobacteria was most abundant on influent wastewater and effluent sample with 57.53% and 77.18% sequence reads, respectively. The class Gammaproteobacteria was highly abundant on influent wastewater with 36.18% sequence reads while it dropped to 3.17% on effluent sample. At the Order level, Enterobacteriales had the sequence reads corresponding to 3.60% in influent wastewater while read dropped to 1.87% in effluent sample. The family Enterobacteriaceae had percentage sequence reads of 2.30% on influent wastewater while effluent sample had sequence reads corresponding to 1.80%. However, at the genus level, the percentage reads corresponding to *Escherichia* subspecies were found to be 0.028% in influent wastewater while the reads in case of effluent sample was 0.811%. Furthermore, the percentage reads for the *Klebsiella* subspecies were 0.038% in influent wastewater and 0.1% in effluent wastewater samples (Table 4).

The *Streptococcus* subspecies are classified under the phylum Firmicutes, class Bacilli, order Lactobacillales and family Streptococcaceae. The percentage sequence reads corresponding to phylum Firmicutes were 16.31% in influent wastewater while the reads were 4.16% in effluent sample. At class level, Bacilli had percentage sequence reads of 7.83 and 2.01% in influent wastewater and effluent sample, respectively. The order Lactobacillales had a read percentage of 7.57% while the read percentage for effluent sample was 1.90%. The family Streptococcaceae had percentage sequence reads of 6.85% in influent wastewater corresponding to second most dominant family on influent while on effluent sample the percentage sequence read was 1.70%. The genus *Streptococcus* was third most dominant in influent wastewater with 6.445% of sequence reads while the percentage sequence read dropped to 0.63% in the effluent sample (Table 5). Similarly, the *Staphylococcus* subspecies are classified under the same phylum, class and order (phylum Firmicutes, class Bacilli, order Lactobacillales) as *Streptococcus* spp., with a different family (Staphylococcaceae). The genus *Staphylococcus* yielded 0.004% of sequence reads in influent wastewater while the percentage sequence read increased to 0.194% in the effluent sample. Furthermore, the genus *Enterococcus* (phylum Firmicutes, class Bacilli, order Lactobacillales, family Enterococcaceae) had percentage reads of 0.063 and 0.037% in the influent sample and effluent sample, respectively.

Pertaining to isolated ESKAPE strains, *Escherichia, Klebsiella* and *Acinetobacter* (resistant to 11 antibiotics, including chloramphenicol, ciprofloxacin, daptomycin, erythromycin, lincomycin, linezolid, nitrofurantoin, penicillin, quinupristin/dalfopristin, tylosin tartrate and vancomycin) have the same AST profiling. The gram-positive cocci, including *Streptococcus* and *Staphylococcus* have resistance to 9 antibiotics (daptomycin, kanamycin, lincomycin, linezolid, nitrofurantoin, penicillin, quinupristin/dalfopristin, tylosin tartrate and vancomycin, also having the same profiling as each other. *Pseudomonas* on the other hand, demonstrated resistance to 7 antibiotics, including Ciprofloxacin, Erythromycin, Daptomycin, Lincomycin, Linezolid, Nitrofurantoin, and Tigecycline. While *Enterococcus* strains were detected in the sequencing reads (Table 5), they were not able to be isolated from the samples after several trials (*N* = 3 replicates ^*^ 3 trials), so the AST profiling was not conducted for them, and will be studied in future works.

Numerous research studies have evidenced the presence of the bacterial community in WW (Szekeres et al., 2017; Zhang et al., 2017; Amorim et al., 2018; Kulkarni et al., 2018). Our previous research study reported the presence of *Pseudomonas* strains with potential drug resistance found in WW for algae cultivation (Limayem et al., 2016). This investigation confirms the predominance of *Pseudomonas* strains including *P. aeruginosa* in WW systems in addition to the identification of some Gram-positive cocci pathogens, namely of *Staphylococcus* genera along with *Gram-negative Klebsiella, Acinetobacter*, and *Escherichia* subspecies and surrogates, with a substantial multidrug resistance requiring urgent intervention. Non-pathogenic bacteria including *Calothrix, Sphingobium*, and *Leptolyngbya* species in addition to some prevalent mutating subspecies such as *Sphingomonas* and *Micrococcus* were recently evidenced with MDR profiling. This finding was particularly relevant for the earlier studies, indicating the presence of drug resistance in both pathogenic and non-pathogenic bacterial strains in the effluent sample. The elucidation of some bacterial isolates, carrying resistant genes are warranted to ascertain that the observed bacterial community requires a targeted nano-treatment such as nanomicelles to eradicate the breeding cycle of resistance and keep beneficial synergism, productivity and the ecological system safe. Future directions will encompass screening of the known and unknown MDR bacteria from WWTP in an effort to reach an efficient intervention and meet the guidelines of water quality standards.

Although results confirmed the existence of six nosocomial strains associated with ESKAPE, including *Pseudomonas* spp., *Streptococcus* spp., *Staphylococcus* spp as well as *Acinetobacter* spp. and *Klebsiella* spp., along with *Escherichia* spp., we elucidated in addition atypical pathogens and non-pathogens strains that were also carrying a broad spectrum of resistance and could be associated to ESKAPE. The non-ESKAPE strains will be further studied for their antimicrobial profiling in our future works since this research study placed emphasis on the evidence of existing ESKAPE and their resistance profiling. Additional statistical analysis including standard deviations from varying conditions will be also addressed in an attempt to trace consistently the MDR breeding ground and meet accurately the water quality standards.

## Data Availability

The raw data supporting the conclusions of this manuscript will be made available by the authors, without undue reservation, to any qualified researcher.

## Author Contributions

AL is the first author on this paper who established the main ideas in this manuscript and articulated and contributed to most of the introduction, the methodologies as well as the discussion sections based on her expertise in the field of microbiology, and drug discovery. SW, MM, and MN mainly collected articles, wrote a few segments of this manuscript. SW contributed to data collection and experimentation. MM contributed heavily to the final revisions and submission of the manuscript. AP majorly contributed to the data presented in the results section. SP formatted and revised the manuscript in accordance with the guidelines provided. BN provided the wastewater samples used in this study and also provided her input on the manuscript. JC participated in the lab experiments.

### Conflict of Interest Statement

The authors declare that the research was conducted in the absence of any commercial or financial relationships that could be construed as a potential conflict of interest.
