# Supplementary material for: High-Throughput Detection of Bacterial Community and Its Drug-Resistance Profiling From Local Reclaimed Wastewater Plants
Source: Front Cell Infect Microbiol. 2019 Oct 4;9:303. doi: 10.3389/fcimb.2019.00303 (PMC6787911; doi:10.3389/fcimb.2019.00303)

**High-throughput Detection of Bacterial Community Structure and its Drug-resistance Profiling from Local Reclaimed Wastewater Plants**

Alya Limayem^1,2^*, Sarah Wasson^1^, Anaya Raj Pokhrel^1^, Jing Chen^1^, Mausam Mehta^3^, Minh Nguyen^4,5^, Shrushti Patil^1^, and Bina Nayak^6^

*^1^ Department of Pharmaceutical Sciences,* Graduate Program, *College of Pharmacy, University of South Florida, 12901 Bruce B. Downs Blvd, MDC 30, Tampa, FL, USA*

*^2^Division of Translational Medicine, Center for Education in Nanobioengineering, University of South Florida, Tampa, FL, USA*

*^3^Morsani College of Medicine, University of South Florida, Tampa, FL, USA*

*^4^College of Public Health, University of South Florida, Tampa, FL, USA*

*^5^College of Arts and Sciences, University of South Florida, Tampa, FL, USA*

*^6^Pinellas County Utilities, Water Quality Division, Largo, FL, USA*

^*^Address correspondence to Alya Limayem, Graduate Programs, Department of Pharmaceutical Sciences, University of South Florida, *12901 Bruce B. Downs Blvd, MDC 30, Tampa, FL 33612*; Tel: (813) 974-7404

E-mail: [alimayem@health.usf.edu](mailto:alimayem@health.usf.edu)

**List of Supplementary Figures**

**Supplementary Figure 1**: Complete list of 1747 species identified in influent and effluent wastewater.

**Supplementary figure 2:** AST profile of MDR isolates identified as *Streptococcus* and *Staphylococcus* sub species.

**Supplementary figure 3:** AST profile of MDR isolate identified as *Pseudomonas* sub species.

**Supplementary figure 4:** AST profile of MDR isolate identified as *Escherichia, Klebsiella* and *Acinetobacter* sub species.

**Supplementary Figure 1**

| Species (N=1747) | untreated-inffluent-wastewater | treated-effluent-wastewater |
| --- | --- | --- |
| ***Abiotrophia defectiva*** | 0 | 10 |
| ***Acetobacterium carbinolicum*** | 107 | 0 |
| ***Acetobacterium fimetarium*** | 3 | 0 |
| ***Acetobacterium malicum*** | 63 | 0 |
| ***Acetobacterium submarinus*** | 39 | 0 |
| ***Acetobacterium tundrae*** | 12 | 0 |
| ***Acetobacterium wieringae*** | 545 | 0 |
| ***Acetobacterium woodii*** | 16 | 0 |
| ***Acholeplasma ales*** | 8 | 0 |
| ***Acholeplasma cavigenitalium*** | 7 | 0 |
| ***Acholeplasma palmae*** | 0 | 1 |
| ***Achromobacter arsenitoxydans*** | 4 | 0 |
| ***Achromobacter insolitus*** | 1 | 0 |
| ***Achromobacter ruhlandii*** | 0 | 1 |
| ***Acidaminobacter hydrogenoformans*** | 276 | 0 |
| ***Acidaminococcus fermentans*** | 131 | 1 |
| ***Acidaminococcus intestini*** | 14 | 0 |
| ***Acidiphilium angustum*** | 4 | 0 |
| ***Acidiphilium symbioticum*** | 0 | 89 |
| ***Acidisoma tundrae*** | 0 | 31 |
| ***Acidithiobacillus albertensis*** | 1 | 0 |
| ***Acidithiobacillus cuprithermicus*** | 1 | 0 |
| ***Acidithiobacillus ferrivorans*** | 1 | 0 |
| ***Acidovorax caeni*** | 60 | 0 |
| ***Acidovorax delafieldii*** | 44 | 0 |
| ***Acidovorax facilis*** | 4 | 0 |
| ***Acidovorax temperans*** | 996 | 1 |
| ***Acidovorax wohlfahrtii*** | 209 | 2 |
| ***Acinetobacter antiviralis*** | 91 | 0 |
| ***Acinetobacter baumannii*** | 0 | 4 |
| ***Acinetobacter baylyi*** | 1 | 0 |
| ***Acinetobacter beijerinckii*** | 15 | 9 |
| ***Acinetobacter bouvetii*** | 66 | 3 |
| ***Acinetobacter gerneri*** | 27 | 147 |
| ***Acinetobacter guillouiae*** | 10 | 546 |
| ***Acinetobacter gyllenbergii*** | 1 | 0 |
| ***Acinetobacter haemolyticus*** | 55 | 0 |
| ***Acinetobacter indicus*** | 72 | 0 |
| ***Acinetobacter johnsonii*** | 347 | 311 |
| ***Acinetobacter junii*** | 4 | 0 |
| ***Acinetobacter lwoffii*** | 7 | 0 |
| ***Acinetobacter marinus*** | 18 | 0 |
| ***Acinetobacter oleivorans*** | 1 | 0 |
| ***Acinetobacter psychrotolerans*** | 136 | 0 |
| ***Acinetobacter radioresistens*** | 3 | 0 |
| ***Acinetobacter rhizosphaerae*** | 3 | 0 |
| ***Acinetobacter schindleri*** | 51 | 8 |
| ***Acinetobacter seohaensis*** | 816 | 14 |
| ***Acinetobacter tjernbergiae*** | 166 | 65 |
| ***Acinetobacter ursingii*** | 0 | 0 |
| ***Acinetobacter xiamenensis*** | 7 | 0 |
| ***Actinoallomurus iriomotensis*** | 0 | 1 |
| ***Actinobacillus capsulatus*** | 1 | 0 |
| ***Actinobacillus parahaemolyticus*** | 0 | 6 |
| ***Actinobacillus pleuropneumoniae*** | 1 | 2 |
| ***Actinobacillus porcinus*** | 5 | 0 |
| ***Actinobacillus rossii*** | 19 | 0 |
| ***Actinobaculum massiliense*** | 382 | 0 |
| ***Actinobaculum suis*** | 15 | 0 |
| ***Actinobaculum urinale*** | 9 | 6 |
| ***Actinocatenispora silicis*** | 1 | 1 |
| ***Actinocatenispora thailandica*** | 3 | 2 |
| ***Actinocorallia herbida*** | 1 | 1 |
| ***Actinokineospora inagensis*** | 4 | 0 |
| ***Actinomadura maheshkhaliensis*** | 0 | 0 |
| ***Actinomyces canis*** | 0 | 0 |
| ***Actinomyces cardiffensis*** | 4 | 1 |
| ***Actinomyces europaeus*** | 0 | 5 |
| ***Actinomyces georgiae*** | 2 | 0 |
| ***Actinomyces lingnae*** | 5 | 13 |
| ***Actinomyces meyeri*** | 0 | 0 |
| ***Actinomyces naturae*** | 106 | 1 |
| ***Actinomyces neuii*** | 3 | 171 |
| ***Actinomyces odontolyticus*** | 7 | 6 |
| ***Actinomyces radingae*** | 0 | 45 |
| ***Actinomyces suimastitidis*** | 4 | 0 |
| ***Actinomyces turicensis*** | 9 | 4 |
| ***Actinopolyspora salina*** | 4 | 0 |
| ***Adlercreutzia equolifaciens*** | 7 | 1 |
| ***Aequorivita lipolytica*** | 10 | 0 |
| ***Aeromicrobium alkaliterrae*** | 0 | 7 |
| ***Aeromicrobium flavum*** | 0 | 0 |
| ***Aeromicrobium ginsengisoli*** | 0 | 4 |
| ***Aeromicrobium kwangyangensis*** | 0 | 4 |
| ***Aeromicrobium marinum*** | 1 | 0 |
| ***Aeromicrobium ponti*** | 0 | 9 |
| ***Aggregatibacter aphrophilus*** | 10 | 0 |
| ***Agrobacterium albertimagni*** | 31 | 0 |
| ***Agrobacterium larrymoorei*** | 13 | 0 |
| ***Agrobacterium tumefaciens*** | 11 | 0 |
| ***Agrobacterium undicola*** | 15 | 1 |
| ***Agrobacterium viscosum*** | 14 | 1 |
| ***Agrococcus versicolor*** | 1 | 0 |
| ***Agromyces salentinus*** | 1 | 1 |
| ***Agromyces succinolyticus*** | 1 | 0 |
| ***Akkermansia muciniphila*** | 188 | 273 |
| ***Alicycliphilus denitrificans*** | 54 | 10 |
| ***Alkalibacillus salilacus*** | 0 | 1 |
| ***Alkalibacterium iburiense*** | 1 | 0 |
| ***Alkaliphilus crotonatoxidans*** | 951 | 13 |
| ***Alkaliphilus metalliredigens*** | 1 | 0 |
| ***Alkaliphilus peptidifermentans*** | 217 | 50 |
| ***Allobaculum stercoricanis*** | 0 | 1 |
| ***Allochromatium palmeri*** | 4 | 0 |
| ***Allochromatium warmingii*** | 1 | 0 |
| ***Alloscardovia omnicolens*** | 3 | 0 |
| ***Amaricoccus kaplicensis*** | 21 | 8 |
| ***Amaricoccus macauensis*** | 13 | 9 |
| ***Amaricoccus tamworthensis*** | 1 | 0 |
| ***Aminiphilus circumscriptus*** | 161 | 1 |
| ***Aminobacter aganoensis*** | 0 | 0 |
| ***Aminobacter aminovorans*** | 1 | 3 |
| ***Aminobacter ciceronei*** | 0 | 1 |
| ***Aminobacterium colombiense*** | 2 | 0 |
| ***Amphritea atlantica*** | 3 | 0 |
| ***Amycolatopsis methanolica*** | 1 | 0 |
| ***Anabaena augstumalis*** | 0 | 2 |
| ***Anaerobacillus alkalilacustre*** | 1 | 3 |
| ***Anaerobranca zavarzinii*** | 34 | 2 |
| ***Anaerococcus lactolyticus*** | 1 | 0 |
| ***Anaerococcus octavius*** | 0 | 2 |
| ***Anaerococcus prevotii*** | 1 | 0 |
| ***Anaerococcus tetradius*** | 1 | 0 |
| ***Anaerococcus vaginalis*** | 1 | 7 |
| ***Anaerofilum pentosovorans*** | 159 | 6 |
| ***Anaerofustis stercorihominis*** | 1 | 0 |
| ***Anaerolinea thermolimosa*** | 1 | 0 |
| ***Anaerolinea thermophila*** | 1 | 0 |
| ***Anaeromusa acidaminophila*** | 19 | 0 |
| ***Anaerovibrio lipolyticus*** | 21 | 0 |
| ***Ancylobacter aquaticus*** | 7 | 0 |
| ***Anoxybacillus ayderensis*** | 0 | 1 |
| ***Aquimarina macrocephali*** | 21 | 0 |
| ***Aquimonas voraii*** | 10 | 0 |
| ***Aquitalea denitrificans*** | 1 | 95 |
| ***Arcanobacterium bernardiae*** | 6 | 1 |
| ***Arcanobacterium phocae*** | 31 | 0 |
| ***Arcanobacterium pluranimalium*** | 53 | 0 |
| ***Arcobacter butzleri*** | 22 | 0 |
| ***Arcobacter cibarius*** | 27 | 0 |
| ***Arcobacter cryaerophilus*** | 3291 | 0 |
| ***Arcobacter defluvii*** | 3611 | 0 |
| ***Arcobacter marinus*** | 81 | 0 |
| ***Arcobacter skirrowii*** | 443 | 0 |
| ***Arcobacter thereius*** | 130 | 0 |
| ***Arenimonas malthae*** | 2 | 0 |
| ***Arthrobacter creatinolyticus*** | 0 | 0 |
| ***Arthrobacter psychrochitiniphilus*** | 0 | 2 |
| ***Arthronema africanum*** | 1 | 0 |
| ***Arthrospira fusiformis*** | 2 | 0 |
| ***Atopobium fossor*** | 1 | 2 |
| ***Atopobium parvulum*** | 1 | 4 |
| ***Aurantimonas coralicida*** | 0 | 3 |
| ***Aurantimonas litoralis*** | 9 | 1 |
| ***Aurantimonas manganoxydans*** | 0 | 6 |
| ***Azoarcus evansii*** | 4 | 9 |
| ***Azohydromonas australica*** | 0 | 0 |
| ***Azomonas insignis*** | 1 | 0 |
| ***Azospira oryzae*** | 56 | 0 |
| ***Azospira restricta*** | 2 | 1 |
| ***Azospirillum palatum*** | 4 | 6 |
| ***Azospirillum rugosum*** | 18 | 12 |
| ***Azovibrio restrictus*** | 32 | 4 |
| ***Bacillus aryabhattai*** | 0 | 27 |
| ***Bacillus asahii*** | 0 | 1 |
| ***Bacillus axarquiensis*** | 0 | 10 |
| ***Bacillus benzoevorans*** | 0 | 2 |
| ***Bacillus butanolivorans*** | 1 | 19 |
| ***Bacillus cereus*** | 0 | 1 |
| ***Bacillus circulans*** | 0 | 6 |
| ***Bacillus coagulans*** | 2 | 0 |
| ***Bacillus deserti*** | 1 | 1 |
| ***Bacillus funiculus*** | 0 | 1 |
| ***Bacillus ginsengihumi*** | 0 | 3 |
| ***Bacillus horneckiae*** | 0 | 6 |
| ***Bacillus humi*** | 0 | 5 |
| ***Bacillus infantis*** | 0 | 5 |
| ***Bacillus lentus*** | 0 | 1 |
| ***Bacillus litoralis*** | 0 | 8 |
| ***Bacillus longiquaesitum*** | 0 | 20 |
| ***Bacillus malacitensis*** | 0 | 3 |
| ***Bacillus mucilaginosus*** | 0 | 12 |
| ***Bacillus muralis*** | 0 | 1 |
| ***Bacillus nealsonii*** | 0 | 16 |
| ***Bacillus niacini*** | 0 | 6 |
| ***Bacillus oleronius*** | 0 | 32 |
| ***Bacillus olivae*** | 0 | 2 |
| ***Bacillus oshimensis*** | 0 | 2 |
| ***Bacillus siralis*** | 0 | 1 |
| ***Bacillus soli*** | 0 | 2 |
| ***Bacillus sonorensis*** | 1 | 5 |
| ***Bacillus thermoamylovorans*** | 0 | 1 |
| ***Bacteriovorax litoralis*** | 9 | 0 |
| ***Bacteriovorax marinus*** | 1 | 0 |
| ***Bacteroides acidifaciens*** | 3 | 8 |
| ***Bacteroides caccae*** | 8 | 12 |
| ***Bacteroides cellulosilyticus*** | 4 | 0 |
| ***Bacteroides clarus*** | 2 | 0 |
| ***Bacteroides coprocola*** | 8 | 88 |
| ***Bacteroides coprophilus*** | 1 | 0 |
| ***Bacteroides denticanum*** | 8 | 27 |
| ***Bacteroides dorei*** | 50 | 10 |
| ***Bacteroides eggerthii*** | 8 | 0 |
| ***Bacteroides finegoldii*** | 7 | 0 |
| ***Bacteroides fragilis*** | 9 | 0 |
| ***Bacteroides gallinarum*** | 0 | 0 |
| ***Bacteroides graminisolvens*** | 5762 | 1 |
| ***Bacteroides intestinalis*** | 2 | 3 |
| ***Bacteroides massiliensis*** | 8 | 11 |
| ***Bacteroides nordii*** | 1 | 0 |
| ***Bacteroides ovatus*** | 18 | 3 |
| ***Bacteroides paurosaccharolyticus*** | 39 | 3 |
| ***Bacteroides plebeius*** | 6 | 49 |
| ***Bacteroides propionicifaciens*** | 4 | 0 |
| ***Bacteroides pyogenes*** | 0 | 8 |
| ***Bacteroides rodentium*** | 27 | 5 |
| ***Bacteroides salyersiae*** | 3 | 0 |
| ***Bacteroides sartorii*** | 71 | 1 |
| ***Bacteroides stercorirosoris*** | 35 | 4 |
| ***Bacteroides stercoris*** | 84 | 38 |
| ***Bacteroides thetaiotaomicron*** | 10 | 22 |
| ***Bacteroides uniformis*** | 17 | 39 |
| ***Bacteroides vulgatus*** | 91 | 181 |
| ***Bacteroides xylanisolvens*** | 218 | 11 |
| ***Bartonella rochalimae*** | 7 | 1 |
| ***Bdellovibrio bacteriovorus*** | 15 | 0 |
| ***Bdellovibrio exovorus*** | 8 | 0 |
| ***Beijerinckia derxii*** | 3 | 8 |
| ***Beijerinckia mobilis*** | 3 | 14 |
| ***Bellilinea caldifistulae*** | 128 | 2 |
| ***Bergeyella zoohelcum*** | 2 | 0 |
| ***Bifidobacterium adolescentis*** | 242 | 116 |
| ***Bifidobacterium angulatum*** | 58 | 9 |
| ***Bifidobacterium animalis*** | 12 | 0 |
| ***Bifidobacterium asteroides*** | 2 | 4 |
| ***Bifidobacterium bifidum*** | 38 | 68 |
| ***Bifidobacterium bombi*** | 70 | 4 |
| ***Bifidobacterium breve*** | 1 | 0 |
| ***Bifidobacterium catenulatum*** | 203 | 37 |
| ***Bifidobacterium choerinum*** | 27 | 3 |
| ***Bifidobacterium cuniculi*** | 0 | 0 |
| ***Bifidobacterium dentium*** | 10 | 2 |
| ***Bifidobacterium gallicum*** | 10 | 6 |
| ***Bifidobacterium indicum*** | 23 | 5 |
| ***Bifidobacterium kashiwanohense*** | 16 | 2 |
| ***Bifidobacterium longum*** | 249 | 68 |
| ***Bifidobacterium merycicum*** | 4 | 0 |
| ***Bifidobacterium pseudolongum*** | 4 | 0 |
| ***Bifidobacterium ruminantium*** | 2 | 1 |
| ***Bifidobacterium saeculare*** | 1 | 0 |
| ***Bifidobacterium scardovii*** | 20 | 4 |
| ***Bifidobacterium stercoris*** | 93 | 57 |
| ***Bifidobacterium thermacidophilum*** | 13 | 0 |
| ***Bifidobacterium thermophilum*** | 1 | 0 |
| ***Bilophila wadsworthia*** | 3 | 0 |
| ***Bizionia saleffrena*** | 1 | 0 |
| ***Blastochloris gulmargensis*** | 1 | 2 |
| ***Blastochloris sulfoviridis*** | 3 | 0 |
| ***Blastococcus aggregatus*** | 1 | 24 |
| ***Blastomonas natatoria*** | 3 | 0 |
| ***Blautia coccoides*** | 173 | 57 |
| ***Blautia glucerasea*** | 2 | 0 |
| ***Blautia hansenii*** | 17 | 37 |
| ***Blautia hydrogenotrophica*** | 9 | 0 |
| ***Blautia obeum*** | 35 | 7 |
| ***Blautia producta*** | 3 | 4 |
| ***Blautia wexlerae*** | 73 | 48 |
| ***Brachybacterium alimentarium*** | 0 | 1 |
| ***Brachybacterium conglomeratum*** | 0 | 9 |
| ***Brachybacterium faecium*** | 1 | 10 |
| ***Brachybacterium squillarum*** | 2 | 1 |
| ***Brachyspira ibaraki*** | 1 | 1 |
| ***Bradyrhizobium cytisi*** | 0 | 8 |
| ***Bradyrhizobium elkanii*** | 3 | 54 |
| ***Bradyrhizobium japonicum*** | 0 | 4 |
| ***Bradyrhizobium jicamae*** | 2 | 42 |
| ***Bradyrhizobium liaoningense*** | 1 | 124 |
| ***Bradyrhizobium pachyrhizi*** | 8 | 279 |
| ***Bradyrhizobium yuanmingense*** | 0 | 0 |
| ***Brevibacillus ginsengisoli*** | 3 | 10 |
| ***Brevibacillus limnophilus*** | 0 | 4 |
| ***Brevibacillus panacihumi*** | 0 | 3 |
| ***Brevibacillus reuszeri*** | 0 | 2 |
| ***Brevibacillus thermoruber*** | 0 | 11 |
| ***Brevibacterium album*** | 1 | 1 |
| ***Brevibacterium aureum*** | 0 | 0 |
| ***Brevibacterium celere*** | 0 | 0 |
| ***Brevibacterium linens*** | 1 | 0 |
| ***Brevundimonas bullata*** | 2 | 3 |
| ***Brevundimonas diminuta*** | 17 | 1 |
| ***Brevundimonas olei*** | 3 | 0 |
| ***Brevundimonas staleyi*** | 1 | 0 |
| ***Brevundimonas terrae*** | 28 | 0 |
| ***Brochothrix thermosphacta*** | 4 | 0 |
| ***Bulleidia moorei*** | 1 | 2 |
| ***Burkholderia acidipaludis*** | 0 | 8 |
| ***Burkholderia brasilensis*** | 3 | 0 |
| ***Burkholderia bryophila*** | 0 | 0 |
| ***Burkholderia cenocepacia*** | 0 | 0 |
| ***Burkholderia lata*** | 3 | 2 |
| ***Burkholderia phenoliruptrix*** | 0 | 4 |
| ***Burkholderia sabiae*** | 0 | 1 |
| ***Burkholderia seminalis*** | 40 | 2 |
| ***Burkholderia ubonensis*** | 36 | 2 |
| ***Burkholderia vietnamiensis*** | 1 | 3 |
| ***Butyricimonas synergistica*** | 0 | 4 |
| ***Butyricimonas virosa*** | 3 | 0 |
| ***Butyrivibrio proteoclasticus*** | 23 | 9 |
| ***Caldanaerobacter hydrothermalis*** | 0 | 1 |
| ***Caldilinea tarbellica*** | 11 | 1 |
| ***Caldithrix palaeochoryensis*** | 1 | 1 |
| ***Caloramator mitchellensis*** | 61 | 1 |
| ***Caloramator uzoniensis*** | 6 | 0 |
| ***Calothrix parietina*** | 241 | 666 |
| ***Campylobacter canadensis*** | 6 | 4 |
| ***Campylobacter concisus*** | 0 | 0 |
| ***Campylobacter curvus*** | 0 | 0 |
| ***Campylobacter gracilis*** | 0 | 6 |
| ***Campylobacter hominis*** | 0 | 10 |
| ***Campylobacter showae*** | 0 | 4 |
| ***Campylobacter ureolyticus*** | 0 | 15 |
| ***Candidatus Amoebophilus asiaticus*** | 92 | 0 |
| ***Candidatus Azobacteroides pseudotrichonymphae*** | 1 | 0 |
| ***Candidatus Blochmannia castaneus*** | 1 | 0 |
| ***Candidatus Blochmannia herculeanus*** | 9 | 0 |
| ***Candidatus Contubernalis alkalaceticum*** | 5 | 0 |
| ***Candidatus Liberibacter africanus*** | 5 | 0 |
| ***Candidatus Liberibacter solanacearum*** | 25 | 0 |
| ***Candidatus Phlomobacter fragariae*** | 1 | 0 |
| ***Candidatus Protochlamydia amoebophila*** | 38 | 2 |
| ***Candidatus Rhabdochlamydia crassificans*** | 1 | 0 |
| ***Candidatus Scalindua brodae*** | 49 | 3 |
| ***Candidatus Tammella caduceiae*** | 236 | 0 |
| ***Capnocytophaga gingivalis*** | 0 | 3 |
| ***Capnocytophaga granulosa*** | 0 | 0 |
| ***Capnocytophaga leadbetteri*** | 0 | 0 |
| ***Carboxydocella ferrireducens*** | 3 | 2 |
| ***Cardiobacterium hominis*** | 0 | 2 |
| ***Carnobacterium gallinarum*** | 1 | 0 |
| ***Carnobacterium inhibens*** | 2 | 1 |
| ***Carnobacterium maltaromaticum*** | 1 | 0 |
| ***Catenibacterium mitsuokai*** | 16 | 0 |
| ***Caulobacter crescentus*** | 2 | 0 |
| ***Caulobacter henricii*** | 1 | 0 |
| ***Caulobacter tundrae*** | 1 | 0 |
| ***Caulobacter vibrioides*** | 3 | 0 |
| ***Cellulomonas oligotrophica*** | 5 | 0 |
| ***Cellulomonas uda*** | 2 | 0 |
| ***Cellulosimicrobium terreum*** | 0 | 0 |
| ***Cellvibrio ostraviensis*** | 0 | 0 |
| ***Cerasicoccus arenae*** | 1 | 0 |
| ***Cetobacterium ceti*** | 17 | 2 |
| ***Cetobacterium somerae*** | 234 | 0 |
| ***Chelatococcus daeguensis*** | 15 | 16 |
| ***Chelonobacter oris*** | 16 | 0 |
| ***Chitinophaga ginsengisoli*** | 0 | 36 |
| ***Chitinophaga soli*** | 17 | 61 |
| ***Chloroflexus aurantiacus*** | 0 | 1 |
| ***Chondromyces pediculatus*** | 3 | 16 |
| ***Chromatium weissei*** | 3 | 0 |
| ***Chromobacterium aquaticum*** | 0 | 15 |
| ***Chromobacterium haemolyticum*** | 1 | 2 |
| ***Chromobacterium piscinae*** | 2 | 2 |
| ***Chromobacterium pseudoviolaceum*** | 2 | 0 |
| ***Chromobacterium subtsugae*** | 19 | 0 |
| ***Chroococcus minutus*** | 4 | 6 |
| ***Chryseobacterium bovis*** | 1 | 0 |
| ***Chryseobacterium caeni*** | 5 | 0 |
| ***Chryseobacterium formosense*** | 30 | 1 |
| ***Chryseobacterium gleum*** | 0 | 1 |
| ***Chryseobacterium hungaricum*** | 0 | 14 |
| ***Chryseobacterium indologenes*** | 0 | 552 |
| ***Chryseobacterium isbiliense*** | 4 | 0 |
| ***Chryseobacterium joostei*** | 0 | 138 |
| ***Chryseobacterium soli*** | 3039 | 0 |
| ***Chryseobacterium taichungense*** | 68 | 5 |
| ***Chthoniobacter flavus*** | 10 | 2 |
| ***Citricoccus alkalitolerans*** | 0 | 150 |
| ***Citricoccus muralis*** | 0 | 17 |
| ***Citrobacter freundii*** | 28 | 6 |
| ***Citrobacter werkmanii*** | 6 | 9 |
| ***Clostridium acetireducens*** | 0 | 1 |
| ***Clostridium akagii*** | 166 | 0 |
| ***Clostridium alkalicellulosi*** | 59 | 17 |
| ***Clostridium baratii*** | 1 | 1 |
| ***Clostridium botulinum*** | 2 | 2 |
| ***Clostridium bovipellis*** | 1 | 0 |
| ***Clostridium butyricum*** | 2 | 0 |
| ***Clostridium cadaveris*** | 105 | 19 |
| ***Clostridium caenicola*** | 16 | 1 |
| ***Clostridium carboxidivorans*** | 1 | 0 |
| ***Clostridium cavendishii*** | 3 | 0 |
| ***Clostridium cellulolyticum*** | 0 | 1 |
| ***Clostridium cellulovorans*** | 1 | 0 |
| ***Clostridium chartatabidum*** | 1 | 0 |
| ***Clostridium chromoreductans*** | 1 | 0 |
| ***Clostridium colicanis*** | 2 | 0 |
| ***Clostridium diolis*** | 0 | 1 |
| ***Clostridium fallax*** | 10 | 2 |
| ***Clostridium frigoris*** | 3 | 2 |
| ***Clostridium hiranonis*** | 18 | 18 |
| ***Clostridium histolyticum*** | 40 | 4 |
| ***Clostridium hveragerdense*** | 2 | 0 |
| ***Clostridium malenominatum*** | 4 | 1 |
| ***Clostridium mesophilum*** | 2 | 0 |
| ***Clostridium nitrophenolicum*** | 1 | 0 |
| ***Clostridium paradoxum*** | 1 | 1 |
| ***Clostridium paraputrificum*** | 6 | 3 |
| ***Clostridium perfringens*** | 23 | 0 |
| ***Clostridium proteolyticus*** | 1 | 0 |
| ***Clostridium saccharobutylicum*** | 2 | 0 |
| ***Clostridium saccharoperbutylacetonicum*** | 7 | 15 |
| ***Clostridium sardiniense*** | 1 | 0 |
| ***Clostridium straminisolvens*** | 14 | 0 |
| ***Clostridium subterminale*** | 0 | 3 |
| ***Clostridium taeniosporum*** | 2 | 0 |
| ***Clostridium tepidiprofundi*** | 2 | 0 |
| ***Clostridium termitidis*** | 7 | 0 |
| ***Clostridium thermoalcaliphilum*** | 19 | 1 |
| ***Clostridium thermosuccinogenes*** | 43 | 9 |
| ***Clostridium thiosulfatireducens*** | 0 | 1 |
| ***Clostridium tunisiense*** | 2 | 0 |
| ***Cohnella damuensis*** | 0 | 1 |
| ***Cohnella hongkongensis*** | 0 | 2 |
| ***Cohnella laeviribosi*** | 0 | 1 |
| ***Cohnella panacarvi*** | 0 | 4 |
| ***Cohnella soli*** | 5 | 3 |
| ***Collinsella aerofaciens*** | 325 | 59 |
| ***Collinsella intestinalis*** | 42 | 3 |
| ***Collinsella tanakaei*** | 20 | 0 |
| ***Comamonas aquatica*** | 3 | 0 |
| ***Comamonas composti*** | 79 | 0 |
| ***Comamonas denitrificans*** | 36 | 0 |
| ***Comamonas kerstersii*** | 352 | 11 |
| ***Comamonas nitrativorans*** | 55 | 0 |
| ***Comamonas odontotermitis*** | 11 | 1 |
| ***Comamonas terrigena*** | 2 | 0 |
| ***Comamonas testosteroni*** | 4 | 0 |
| ***Conchiformibius kuhniae*** | 1 | 2 |
| ***Conexibacter woesei*** | 1 | 0 |
| ***Coprobacillus cateniformis*** | 0 | 17 |
| ***Coprococcus catus*** | 3 | 0 |
| ***Coprococcus eutactus*** | 2 | 28 |
| ***Coraliomargarita akajimensis*** | 19 | 1 |
| ***Corynebacterium accolens*** | 0 | 2 |
| ***Corynebacterium acetoacidophilum*** | 0 | 1 |
| ***Corynebacterium amycolatum*** | 4 | 2 |
| ***Corynebacterium appendicis*** | 0 | 0 |
| ***Corynebacterium argentoratense*** | 0 | 0 |
| ***Corynebacterium atypicum*** | 1 | 204 |
| ***Corynebacterium auriscanis*** | 0 | 0 |
| ***Corynebacterium bovis*** | 0 | 0 |
| ***Corynebacterium callunae*** | 1 | 0 |
| ***Corynebacterium canis*** | 0 | 1 |
| ***Corynebacterium coyleae*** | 0 | 6 |
| ***Corynebacterium diphtheriae*** | 9 | 0 |
| ***Corynebacterium durum*** | 1 | 6 |
| ***Corynebacterium efficiens*** | 0 | 2 |
| ***Corynebacterium falsenii*** | 2 | 0 |
| ***Corynebacterium flavescens*** | 4 | 0 |
| ***Corynebacterium genitalium*** | 1 | 3 |
| ***Corynebacterium glucuronolyticum*** | 0 | 68 |
| ***Corynebacterium hansenii*** | 4 | 0 |
| ***Corynebacterium kroppenstedtii*** | 2 | 11 |
| ***Corynebacterium kutscheri*** | 5 | 4 |
| ***Corynebacterium lipophiloflavum*** | 1 | 0 |
| ***Corynebacterium mastitidis*** | 0 | 5 |
| ***Corynebacterium matruchotii*** | 0 | 4 |
| ***Corynebacterium minutissimum*** | 0 | 1 |
| ***Corynebacterium mycetoides*** | 0 | 1 |
| ***Corynebacterium nuruki*** | 3 | 3 |
| ***Corynebacterium pseudogenitalium*** | 0 | 0 |
| ***Corynebacterium pyruviciproducens*** | 0 | 3 |
| ***Corynebacterium simulans*** | 2 | 0 |
| ***Corynebacterium striatum*** | 1 | 0 |
| ***Corynebacterium testudinoris*** | 1 | 0 |
| ***Corynebacterium timonense*** | 0 | 2 |
| ***Corynebacterium tuberculostearicum*** | 3 | 29 |
| ***Corynebacterium ulceribovis*** | 0 | 0 |
| ***Corynebacterium urealyticum*** | 1 | 0 |
| ***Corynebacterium ureicelerivorans*** | 0 | 6 |
| ***Corynebacterium variabile*** | 27 | 0 |
| ***Corynebacterium vitaeruminis*** | 4 | 11 |
| ***Corynebacterium xerosis*** | 51 | 0 |
| ***Cryocola antiquus*** | 0 | 1 |
| ***Cupriavidus metallidurans*** | 0 | 4 |
| ***Cupriavidus pauculus*** | 0 | 2 |
| ***Curtobacterium albidum*** | 2 | 1 |
| ***Curtobacterium pusillum*** | 8 | 0 |
| ***Curvibacter gracilis*** | 42 | 6 |
| ***Curvibacter lanceolatus*** | 6 | 0 |
| ***Cyanobacterium aponinum*** | 0 | 43 |
| ***Cycloclasticus oligotrophus*** | 2 | 0 |
| ***Dactylosporangium maewongense*** | 1 | 3 |
| ***Dechloromonas agitata*** | 62 | 0 |
| ***Dechloromonas aromatica*** | 53 | 1 |
| ***Dechloromonas fungiphilus*** | 33 | 0 |
| ***Dechloromonas hortensis*** | 224 | 3 |
| ***Deefgea chitinilytica*** | 1 | 0 |
| ***Deferribacter autotrophicus*** | 2 | 3 |
| ***Deinococcus aerius*** | 0 | 3 |
| ***Deinococcus aetherius*** | 0 | 1 |
| ***Delftia acidovorans*** | 0 | 0 |
| ***Delftia lacustris*** | 3 | 76 |
| ***Delftia tsuruhatensis*** | 1 | 4 |
| ***Demequina aurantiaca*** | 1 | 0 |
| ***Demequina globuliformis*** | 7 | 0 |
| ***Denitratisoma oestradiolicum*** | 4 | 0 |
| ***Denitrobacter permanens*** | 4 | 0 |
| ***Dermacoccus abyssi*** | 0 | 1 |
| ***Dermacoccus nishinomiyaensis*** | 1 | 0 |
| ***Dermatophilus congolensis*** | 13 | 0 |
| ***Desulfacinum subterraneum*** | 1 | 0 |
| ***Desulfitobacterium chlororespirans*** | 0 | 2 |
| ***Desulfobacter hydrogenophilus*** | 2 | 0 |
| ***Desulfobacter latus*** | 1 | 0 |
| ***Desulfobacter postgatei*** | 43 | 0 |
| ***Desulfobacter vibrioformis*** | 5 | 0 |
| ***Desulfobulbus elongatus*** | 119 | 0 |
| ***Desulfobulbus propionicus*** | 30 | 0 |
| ***Desulfobulbus rhabdoformis*** | 110 | 0 |
| ***Desulfocapsa sulfexigens*** | 1 | 0 |
| ***Desulfofaba gelida*** | 7 | 0 |
| ***Desulfofrigus oceanense*** | 1 | 1 |
| ***Desulfomicrobium baculatum*** | 406 | 0 |
| ***Desulfomicrobium escambiense*** | 42 | 0 |
| ***Desulfomicrobium macestii*** | 4 | 0 |
| ***Desulfomicrobium norvegicum*** | 874 | 0 |
| ***Desulfomonile tiedjei*** | 16 | 0 |
| ***Desulfonatronum thiosulfatophilum*** | 15 | 1 |
| ***Desulfonauticus autotrophicus*** | 75 | 0 |
| ***Desulfonauticus submarinus*** | 128 | 0 |
| ***Desulfosarcina cetonica*** | 197 | 0 |
| ***Desulfosarcina ovata*** | 48 | 0 |
| ***Desulfosarcina variabilis*** | 14 | 0 |
| ***Desulfosporomusa polytropa*** | 0 | 0 |
| ***Desulfosporosinus acidiphilus*** | 0 | 6 |
| ***Desulfosporosinus auripigmenti*** | 0 | 10 |
| ***Desulfosporosinus lacus*** | 6 | 0 |
| ***Desulfosporosinus meridiei*** | 0 | 2 |
| ***Desulfotalea arctica*** | 13 | 0 |
| ***Desulfotignum phosphitoxidans*** | 97 | 0 |
| ***Desulfotomaculum indicum*** | 53 | 1 |
| ***Desulfotomaculum nigrificans*** | 0 | 2 |
| ***Desulfovibrio aceae*** | 2 | 0 |
| ***Desulfovibrio alcoholivorans*** | 7 | 0 |
| ***Desulfovibrio aminophilus*** | 1 | 0 |
| ***Desulfovibrio burkinensis*** | 22 | 0 |
| ***Desulfovibrio butyratiphilus*** | 10 | 0 |
| ***Desulfovibrio caledoniensis*** | 1 | 0 |
| ***Desulfovibrio carbinolicus*** | 3 | 0 |
| ***Desulfovibrio carbinoliphilus*** | 1 | 0 |
| ***Desulfovibrio cuneatus*** | 12 | 0 |
| ***Desulfovibrio desulfuricans*** | 255 | 0 |
| ***Desulfovibrio fairfieldensis*** | 74 | 1 |
| ***Desulfovibrio ferrireducens*** | 3 | 0 |
| ***Desulfovibrio ferrophilus*** | 1 | 0 |
| ***Desulfovibrio frigidus*** | 12 | 0 |
| ***Desulfovibrio idahonensis*** | 5 | 0 |
| ***Desulfovibrio intestinalis*** | 166 | 0 |
| ***Desulfovibrio litoralis*** | 27 | 0 |
| ***Desulfovibrio magneticus*** | 21 | 0 |
| ***Desulfovibrio marinus*** | 3 | 0 |
| ***Desulfovibrio marrakechensis*** | 2 | 0 |
| ***Desulfovibrio mexicanus*** | 38 | 0 |
| ***Desulfovibrio oryzae*** | 87 | 2 |
| ***Desulfovibrio piger*** | 156 | 0 |
| ***Desulfovibrio psychrotolerans*** | 7 | 0 |
| ***Desulfovibrio putealis*** | 3 | 0 |
| ***Desulfovibrio salexigens*** | 1 | 0 |
| ***Desulfovibrio simplex*** | 69 | 0 |
| ***Desulfovibrio sulfodismutans*** | 11 | 0 |
| ***Desulfovibrio tunisiensis*** | 1 | 0 |
| ***Desulfovibrio vulgaris*** | 42 | 0 |
| ***Desulfurispirillum alkaliphilum*** | 0 | 1 |
| ***Desulfurispora thermophila*** | 132 | 1 |
| ***Desulfuromonas svalbardensis*** | 2 | 2 |
| ***Desulfuromonas thiophila*** | 4 | 0 |
| ***Desulfuromusa succinoxidans*** | 1 | 0 |
| ***Devosia geojensis*** | 0 | 2 |
| ***Devosia ginsengisoli*** | 0 | 0 |
| ***Devosia insulae*** | 0 | 3 |
| ***Dialister invisus*** | 53 | 8 |
| ***Diaphorobacter nitroreducens*** | 10 | 0 |
| ***Dickeya paradisiaca*** | 0 | 0 |
| ***Dietzia alimentaria*** | 2 | 5 |
| ***Dietzia cercidiphylli*** | 0 | 1 |
| ***Dietzia cinnamea*** | 0 | 2 |
| ***Dietzia maris*** | 2 | 6 |
| ***Dietzia natronolimnaea*** | 0 | 4 |
| ***Dokdonella fugitiva*** | 20 | 6 |
| ***Dorea formicigenerans*** | 19 | 0 |
| ***Dyadobacter alkalitolerans*** | 1 | 0 |
| ***Dyadobacter hamtensis*** | 1 | 0 |
| ***Dysgonomonas capnocytophagoides*** | 2 | 0 |
| ***Dysgonomonas gadei*** | 4 | 0 |
| ***Dysgonomonas hofstadii*** | 3 | 0 |
| ***Dysgonomonas mossii*** | 27 | 0 |
| ***Dysgonomonas wimpennyi*** | 112 | 9 |
| ***Ectothiorhodospira haloalkaliphila*** | 11 | 18 |
| ***Ectothiorhodospira imhoffii*** | 3 | 0 |
| ***Edaphobacter modestus*** | 3 | 8 |
| ***Eggerthella lenta*** | 18 | 2 |
| ***Eggerthella sinensis*** | 36 | 0 |
| ***Ehrlichia ovina*** | 3 | 8 |
| ***Eikenella corrodens*** | 309 | 0 |
| ***Elizabethkingia meningoseptica*** | 1 | 0 |
| ***Emticicia oligotrophica*** | 50 | 2 |
| ***Enhydrobacter aerosaccus*** | 41 | 3 |
| ***Enterobacter aceae*** | 3 | 4 |
| ***Enterobacter aerogenes*** | 3 | 2 |
| ***Enterobacter amnigenus*** | 15 | 64 |
| ***Enterobacter asburiae*** | 4 | 81 |
| ***Enterobacter cancerogenus*** | 0 | 1 |
| ***Enterobacter cloacae*** | 4 | 32 |
| ***Enterobacter hormaechei*** | 29 | 132 |
| ***Enterobacter ludwigii*** | 1 | 6 |
| ***Enterobacter nickellidurans*** | 7 | 24 |
| ***Enterobacter soli*** | 6 | 17 |
| ***Enterococcus aquimarinus*** | 17 | 0 |
| ***Enterococcus asini*** | 5 | 0 |
| ***Enterococcus avium*** | 10 | 3 |
| ***Enterococcus camelliae*** | 1 | 0 |
| ***Enterococcus casseliflavus*** | 6 | 0 |
| ***Enterococcus cecorum*** | 10 | 0 |
| ***Enterococcus durans*** | 4 | 3 |
| ***Enterococcus faecalis*** | 3 | 47 |
| ***Enterococcus faecium*** | 1 | 0 |
| ***Enterococcus gallinarum*** | 3 | 0 |
| ***Enterococcus gilvus*** | 13 | 0 |
| ***Enterococcus hawaiiensis*** | 3 | 0 |
| ***Enterococcus inusitatus*** | 3 | 0 |
| ***Enterococcus italicus*** | 12 | 0 |
| ***Enterococcus lactis*** | 13 | 2 |
| ***Enterococcus mundtii*** | 2 | 0 |
| ***Enterococcus rottae*** | 3 | 0 |
| ***Enterococcus silesiacus*** | 14 | 0 |
| ***Enterococcus sulfureus*** | 1 | 0 |
| ***Erwinia billingiae*** | 1 | 5 |
| ***Erwinia mallotivora*** | 0 | 32 |
| ***Erwinia oleae*** | 1 | 0 |
| ***Erwinia papayae*** | 0 | 4 |
| ***Erwinia psidii*** | 1 | 0 |
| ***Erwinia pyrifoliae*** | 0 | 1 |
| ***Erwinia tasmaniensis*** | 0 | 2 |
| ***Erysipelothrix inopinata*** | 25 | 3 |
| ***Erysipelothrix muris*** | 54 | 14 |
| ***Erysipelothrix rhusiopathiae*** | 1 | 0 |
| ***Erythrobacter aquimaris*** | 6 | 7 |
| ***Erythromicrobium ramosum*** | 2 | 0 |
| ***Escherichia albertii*** | 50 | 1186 |
| ***Escherichia coli*** | 4 | 33 |
| ***Eubacterium biforme*** | 117 | 0 |
| ***Eubacterium callanderi*** | 7 | 0 |
| ***Eubacterium cylindroides*** | 14 | 0 |
| ***Eubacterium dolichum*** | 8 | 14 |
| ***Eubacterium limosum*** | 2 | 0 |
| ***Euzebya tangerina*** | 1 | 2 |
| ***Facklamia hominis*** | 8 | 0 |
| ***Facklamia tabacinasalis*** | 2 | 0 |
| ***Faecalibacterium prausnitzii*** | 157 | 160 |
| ***Ferrimonas futtsuensis*** | 7 | 0 |
| ***Fervidobacterium pennivorans*** | 1 | 0 |
| ***Fibrobacter succinogenes*** | 1 | 0 |
| ***Filifactor villosus*** | 1 | 0 |
| ***Finegoldia magna*** | 6 | 333 |
| ***Flavisolibacter ginsengisoli*** | 2 | 1 |
| ***Flavobacterium antarcticum*** | 1 | 0 |
| ***Flavobacterium aquatile*** | 1 | 0 |
| ***Flavobacterium cheniae*** | 983 | 0 |
| ***Flavobacterium chungangense*** | 1 | 0 |
| ***Flavobacterium cucumis*** | 5 | 0 |
| ***Flavobacterium flevense*** | 80 | 0 |
| ***Flavobacterium gelidilacus*** | 0 | 0 |
| ***Flavobacterium kamogawaensis*** | 13 | 0 |
| ***Flavobacterium micromati*** | 4 | 0 |
| ***Flavobacterium reichenbachii*** | 89 | 0 |
| ***Flavobacterium saliperosum*** | 49 | 0 |
| ***Flavobacterium succinicans*** | 15 | 0 |
| ***Flavobacterium suncheonense*** | 1 | 0 |
| ***Flavobacterium terrigena*** | 149 | 1 |
| ***Flavobacterium weaverense*** | 24 | 0 |
| ***Frankia alni*** | 2 | 1 |
| ***Friedmanniella antarctica*** | 0 | 6 |
| ***Friedmanniella okinawensis*** | 0 | 2 |
| ***Fructobacillus pseudoficulneus*** | 1 | 0 |
| ***Fusibacter paucivorans*** | 1 | 0 |
| ***Fusobacterium canifelinum*** | 0 | 4 |
| ***Fusobacterium gonidiaformans*** | 47 | 9 |
| ***Fusobacterium naviforme*** | 3 | 14 |
| ***Fusobacterium nucleatum*** | 0 | 10 |
| ***Fusobacterium periodonticum*** | 0 | 16 |
| ***Fusobacterium simiae*** | 1 | 0 |
| ***Gallibacterium melopsittaci*** | 2 | 0 |
| ***Gallionella ferruginea*** | 2 | 8 |
| ***Gardnerella vaginalis*** | 9 | 25 |
| ***Gemella cunicula*** | 6 | 11 |
| ***Gemella haemolysans*** | 0 | 23 |
| ***Gemella sanguinis*** | 0 | 26 |
| ***Gemmata obscuriglobus*** | 1 | 0 |
| ***Gemmatimonas aurantiaca*** | 0 | 2 |
| ***Geobacillus anatolicus*** | 1 | 0 |
| ***Geobacillus gargensis*** | 1 | 0 |
| ***Geobacillus thermoglucosidans*** | 10 | 7 |
| ***Geobacillus toebii*** | 0 | 9 |
| ***Geobacillus vulcani*** | 0 | 0 |
| ***Geobacter chapelleii*** | 7 | 0 |
| ***Geobacter lovleyi*** | 24 | 0 |
| ***Geobacter metallireducens*** | 1 | 0 |
| ***Geobacter pickeringii*** | 9 | 0 |
| ***Geobacter thiogenes*** | 10 | 0 |
| ***Geobacter toluenoxydans*** | 5 | 0 |
| ***Geobacter uraniireducens*** | 1 | 0 |
| ***Georgenia ferrireducens*** | 62 | 1 |
| ***Georgenia halophila*** | 4 | 0 |
| ***Geothrix fermentans*** | 33 | 0 |
| ***Geovibrio ferrireducens*** | 1 | 0 |
| ***Gloeotrichia echinulata*** | 0 | 96 |
| ***Gluconobacter japonicus*** | 1 | 0 |
| ***Gluconobacter kondonii*** | 1 | 1 |
| ***Gluconobacter krungthepensis*** | 27 | 6 |
| ***Gluconobacter morbifer*** | 23 | 15 |
| ***Gluconobacter nephelii*** | 0 | 1 |
| ***Gluconobacter thailandicus*** | 0 | 0 |
| ***Gordonia amarae*** | 14 | 0 |
| ***Gordonia australis*** | 2 | 0 |
| ***Gordonia cholesterolivorans*** | 5 | 0 |
| ***Gordonia effusa*** | 1 | 0 |
| ***Gordonia hirsuta*** | 48 | 0 |
| ***Gordonia polyisoprenivorans*** | 2 | 0 |
| ***Gordonia soli*** | 2 | 0 |
| ***Gordonia terrae*** | 3 | 0 |
| ***Gordonia westfalica*** | 9 | 0 |
| ***Granulicatella adiacens*** | 2 | 28 |
| ***Granulicatella elegans*** | 0 | 0 |
| ***Haemophilus haemolyticus*** | 0 | 2 |
| ***Haemophilus parainfluenzae*** | 3 | 35 |
| ***Haemophilus quentini*** | 0 | 0 |
| ***Halanaerobium alcaliphilum*** | 8 | 0 |
| ***Halanaerobium fermentans*** | 0 | 1 |
| ***Haliscomenobacter hydrossis*** | 3 | 0 |
| ***Halochromatium salexigens*** | 1 | 0 |
| ***Halomonas maura*** | 2 | 0 |
| ***Halomonas nitritophilus*** | 0 | 1 |
| ***Halomonas sinaiensis*** | 1 | 0 |
| ***Halorhodospira halochloris*** | 1 | 1 |
| ***Halothiobacillus neapolitanus*** | 86 | 0 |
| ***Helicobacter bilis*** | 0 | 1 |
| ***Helicobacter cinaedi*** | 0 | 3 |
| ***Helicobacter mastomyrinus*** | 1 | 0 |
| ***Helicobacter suncus*** | 2 | 0 |
| ***Heliorestis baculata*** | 520 | 0 |
| ***Herbaspirillum aquaticum*** | 1 | 0 |
| ***Herbaspirillum chlorophenolicum*** | 0 | 2 |
| ***Herbaspirillum huttiense*** | 0 | 5 |
| ***Herbaspirillum magnetovibrio*** | 8 | 0 |
| ***Hirschia baltica*** | 2 | 0 |
| ***Hydrocarboniphaga daqingensis*** | 1 | 1 |
| ***Hydrocoleum lyngbyaceum*** | 0 | 1 |
| ***Hydrogenophaga caeni*** | 2 | 0 |
| ***Hydrogenophaga defluvii*** | 60 | 1 |
| ***Hydrogenophaga pseudoflava*** | 6 | 0 |
| ***Hydrogenophilus denitrificans*** | 2 | 0 |
| ***Hydrogenophilus halorhabdus*** | 1 | 0 |
| ***Hydrogenophilus hirschii*** | 5 | 0 |
| ***Hymenobacter aerophilus*** | 0 | 2 |
| ***Hymenobacter xinjiangensis*** | 1 | 0 |
| ***Hyphomicrobium aestuarii*** | 2 | 7 |
| ***Hyphomicrobium vulgare*** | 2 | 1 |
| ***Hyphomicrobium zavarzinii*** | 3 | 0 |
| ***Hyphomonas hirschiana*** | 1 | 0 |
| ***Hyphomonas oceanitis*** | 1 | 0 |
| ***Inquilinus ginsengisoli*** | 1 | 0 |
| ***Isosphaera pallida*** | 2 | 0 |
| ***Janibacter anophelis*** | 2 | 2 |
| ***Janibacter hoylei*** | 1 | 0 |
| ***Jannaschia seohaensis*** | 9 | 2 |
| ***Janthinobacterium agaricidamnosum*** | 1314 | 22 |
| ***Janthinobacterium lividum*** | 3792 | 3 |
| ***Jiangella alkaliphila*** | 3 | 0 |
| ***Johnsonella ignava*** | 6 | 12 |
| ***Jonesia quinghaiensis*** | 0 | 0 |
| ***Kaistia adipata*** | 2 | 0 |
| ***Kaistobacter terrae*** | 1 | 4 |
| ***Kineococcus gynurae*** | 0 | 1 |
| ***Kineosporia mikuniensis*** | 1 | 9 |
| ***Kitasatospora melanogena*** | 1 | 0 |
| ***Klebsiella granulomatis*** | 12 | 28 |
| ***Klebsiella oxytoca*** | 34 | 22 |
| ***Klebsiella pneumoniae*** | 9 | 27 |
| ***Klebsiella variicola*** | 19 | 74 |
| ***Knoellia aerolata*** | 13 | 0 |
| ***Knoellia subterranea*** | 0 | 2 |
| ***Kocuria assamensis*** | 0 | 2 |
| ***Kocuria gwangalliensis*** | 2 | 44 |
| ***Kocuria koreensis*** | 0 | 0 |
| ***Kocuria palustris*** | 0 | 2 |
| ***Kocuria rhizophila*** | 5 | 5 |
| ***Kocuria rosea*** | 1 | 0 |
| ***Kosmotoga arenicorallina*** | 0 | 0 |
| ***Kouleothrix aurantiaca*** | 0 | 1 |
| ***Kribbella ginsengisoli*** | 2 | 1 |
| ***Kurthia gibsonii*** | 0 | 1 |
| ***Kushneria aurantia*** | 1 | 0 |
| ***Kushneria avicenniae*** | 0 | 0 |
| ***Kushneria indalinina*** | 5 | 8 |
| ***Kytococcus sedentarius*** | 1 | 0 |
| ***Labrys wisconsinensis*** | 4 | 2 |
| ***Laceyella putida*** | 0 | 1 |
| ***Lachnospira pectinoschiza*** | 31 | 69 |
| ***Lactobacillus acidophilus*** | 5 | 1 |
| ***Lactobacillus agilis*** | 2 | 0 |
| ***Lactobacillus antri*** | 3 | 0 |
| ***Lactobacillus apis*** | 0 | 0 |
| ***Lactobacillus camelliae*** | 2 | 0 |
| ***Lactobacillus casei*** | 2 | 0 |
| ***Lactobacillus crispatus*** | 0 | 5 |
| ***Lactobacillus crustorum*** | 1 | 0 |
| ***Lactobacillus delbrueckii*** | 16 | 0 |
| ***Lactobacillus equi*** | 1 | 0 |
| ***Lactobacillus equicursoris*** | 4 | 0 |
| ***Lactobacillus fermentum*** | 3 | 0 |
| ***Lactobacillus gasseri*** | 10 | 0 |
| ***Lactobacillus gigeriorum*** | 1 | 0 |
| ***Lactobacillus hayakitensis*** | 8 | 0 |
| ***Lactobacillus helveticus*** | 0 | 2 |
| ***Lactobacillus iners*** | 0 | 72 |
| ***Lactobacillus ingluviei*** | 2 | 0 |
| ***Lactobacillus intermedius*** | 2 | 0 |
| ***Lactobacillus intestinalis*** | 1 | 2 |
| ***Lactobacillus japonicus*** | 2 | 0 |
| ***Lactobacillus jensenii*** | 0 | 3 |
| ***Lactobacillus johnsonii*** | 6 | 0 |
| ***Lactobacillus letivazi*** | 28 | 0 |
| ***Lactobacillus manihotivorans*** | 1 | 0 |
| ***Lactobacillus mucosae*** | 8 | 0 |
| ***Lactobacillus oris*** | 1 | 0 |
| ***Lactobacillus panis*** | 1 | 0 |
| ***Lactobacillus parabuchneri*** | 1 | 0 |
| ***Lactobacillus paracasei*** | 1 | 0 |
| ***Lactobacillus parakefiri*** | 1 | 0 |
| ***Lactobacillus pontis*** | 2 | 0 |
| ***Lactobacillus reuteri*** | 8 | 1 |
| ***Lactobacillus rhamnosus*** | 3 | 0 |
| ***Lactobacillus ruminis*** | 8 | 0 |
| ***Lactobacillus salivarius*** | 17 | 0 |
| ***Lactobacillus sanfranciscensis*** | 2 | 0 |
| ***Lactobacillus secaliphilus*** | 1 | 0 |
| ***Lactobacillus senmaizukei*** | 1 | 0 |
| ***Lactobacillus siliginis*** | 2 | 0 |
| ***Lactobacillus similis*** | 15 | 0 |
| ***Lactobacillus taiwanensis*** | 1 | 4 |
| ***Lactobacillus ultunensis*** | 3 | 1 |
| ***Lactobacillus vaginalis*** | 8 | 0 |
| ***Lactobacillus versmoldensis*** | 1 | 0 |
| ***Lactobacillus zeae*** | 1 | 0 |
| ***Lactococcus fujiensis*** | 3 | 1 |
| ***Lactococcus garvieae*** | 6 | 0 |
| ***Lactococcus lactis*** | 3 | 0 |
| ***Lactococcus raffinolactis*** | 54 | 0 |
| ***Lampropedia hyalina*** | 1 | 0 |
| ***Lautropia mirabilis*** | 3 | 4 |
| ***Legionella fallonii*** | 0 | 3 |
| ***Legionella moravica*** | 1 | 0 |
| ***Legionella pneumophila*** | 1 | 0 |
| ***Legionella rowbothamii*** | 1 | 0 |
| ***Legionella shakespearei*** | 6 | 0 |
| ***Legionella waltersii*** | 1 | 0 |
| ***Lentibacillus salinarum*** | 2 | 0 |
| ***Lentzea flavoverrucoides*** | 0 | 2 |
| ***Leptolyngbya antarctica*** | 1 | 1 |
| ***Leptolyngbya laminosa*** | 16 | 6535 |
| ***Leptospira licerasiae*** | 2 | 0 |
| ***Leptothrix discophora*** | 20 | 6 |
| ***Leptotrichia buccalis*** | 0 | 0 |
| ***Leptotrichia goodfellowii*** | 3 | 0 |
| ***Leptotrichia shahii*** | 0 | 6 |
| ***Leptotrichia trevisanii*** | 0 | 1 |
| ***Leptotrichia wadei*** | 0 | 3 |
| ***Leucobacter chironomi*** | 33 | 8 |
| ***Leucobacter chromiiresistens*** | 2 | 0 |
| ***Leucobacter iarius*** | 1 | 0 |
| ***Leucobacter komagatae*** | 15 | 0 |
| ***Leucobacter luti*** | 5 | 0 |
| ***Leuconostoc citreum*** | 5 | 0 |
| ***Leuconostoc gasicomitatum*** | 2 | 0 |
| ***Leuconostoc gelidum*** | 1 | 0 |
| ***Leuconostoc lactis*** | 4 | 0 |
| ***Leuconostoc mesenteroides*** | 1 | 0 |
| ***Leuconostoc pseudomesenteroides*** | 2 | 0 |
| ***Leucothrix mucor*** | 1 | 2 |
| ***Lewinella lutea*** | 1 | 2 |
| ***Lewinella marina*** | 10 | 0 |
| ***Limnobacter litoralis*** | 12 | 1 |
| ***Limnohabitans parvus*** | 2 | 0 |
| ***Limnohabitans planktonicus*** | 2 | 0 |
| ***Litoricola lipolytica*** | 1 | 0 |
| ***Loktanella agnita*** | 0 | 1 |
| ***Longilinea arvoryzae*** | 39 | 2 |
| ***Luteibacter anthropi*** | 3 | 0 |
| ***Luteimonas aquatica*** | 25 | 0 |
| ***Luteimonas composti*** | 2 | 0 |
| ***Luteimonas lutimaris*** | 2 | 0 |
| ***Luteimonas terricola*** | 4 | 0 |
| ***Luteococcus peritonei*** | 25 | 2 |
| ***Luteolibacter algae*** | 8 | 4 |
| ***Lutibacterium anuloederans*** | 0 | 2 |
| ***Lysinibacillus boronitolerans*** | 0 | 5 |
| ***Lysinibacillus parviboronicapiens*** | 0 | 1 |
| ***Lysinibacillus xylanilyticus*** | 0 | 15 |
| ***Lysobacter daejeonensis*** | 0 | 0 |
| ***Lysobacter enzymogenes*** | 0 | 0 |
| ***Lysobacter yangpyeongensis*** | 1 | 0 |
| ***Macrococcus bovicus*** | 1 | 0 |
| ***Magnetospirillum bellicus*** | 1 | 0 |
| ***Magnetospirillum magnetotacticum*** | 0 | 10 |
| ***Mannheimia caviae*** | 2 | 2 |
| ***Maricaulis indicus*** | 4 | 868 |
| ***Marichromatium gracile*** | 2 | 2 |
| ***Marinitoga hydrogenitolerans*** | 3 | 0 |
| ***Marinitoga okinawensis*** | 1 | 0 |
| ***Marinobacter arcticus*** | 0 | 0 |
| ***Marinobacter bacchus*** | 1 | 0 |
| ***Marinobacter santoriniensis*** | 2 | 0 |
| ***Marinobacter szutsaonensis*** | 1 | 0 |
| ***Marinomonas arctica*** | 2 | 0 |
| ***Marinomonas basaltis*** | 7 | 0 |
| ***Marinomonas brasiliensis*** | 4 | 0 |
| ***Marinomonas foliarum*** | 3 | 0 |
| ***Mechercharimyces asporophorigenens*** | 1 | 3 |
| ***Megamonas funiformis*** | 8 | 11 |
| ***Megasphaera elsdenii*** | 35 | 3 |
| ***Megasphaera hominis*** | 29 | 12 |
| ***Megasphaera paucivorans*** | 17 | 0 |
| ***Mesoplasma entomophilum*** | 1 | 14 |
| ***Mesorhizobium camelthorni*** | 5 | 1 |
| ***Mesorhizobium huakuii*** | 16 | 0 |
| ***Mesorhizobium opportunistum*** | 3 | 0 |
| ***Mesorhizobium septentrionale*** | 8 | 1 |
| ***Methanobacterium beijingense*** | 1 | 0 |
| ***Methanobacterium bryantii*** | 1 | 0 |
| ***Methanobrevibacter arboriphilus*** | 7 | 0 |
| ***Methanobrevibacter smithii*** | 25 | 0 |
| ***Methanofollis ethanolicus*** | 1 | 0 |
| ***Methanosaeta concilii*** | 56 | 0 |
| ***Methanosphaera stadtmanae*** | 2 | 0 |
| ***Methylobacillus flagellatus*** | 1 | 0 |
| ***Methylobacillus glycogenes*** | 18 | 3 |
| ***Methylobacterium adhaesivum*** | 0 | 393 |
| ***Methylobacterium goesingense*** | 75 | 760 |
| ***Methylobacterium gregans*** | 0 | 8 |
| ***Methylobacterium hispanicum*** | 0 | 8 |
| ***Methylobacterium komagatae*** | 0 | 12 |
| ***Methylobacterium longum*** | 1 | 7 |
| ***Methylobacterium marchantiae*** | 46 | 6 |
| ***Methylobacterium mesophilicum*** | 1 | 57 |
| ***Methylobacterium organophilum*** | 1 | 16 |
| ***Methylobacterium persicinum*** | 0 | 0 |
| ***Methylobacterium radiotolerans*** | 1 | 4 |
| ***Methylocaldum gracile*** | 1 | 0 |
| ***Methylocaldum tepidum*** | 1 | 0 |
| ***Methylocella silvestris*** | 1 | 0 |
| ***Methylonatrum kenyense*** | 0 | 5 |
| ***Methylophaga lonarensis*** | 2 | 0 |
| ***Methylosinus pucelana*** | 10 | 3 |
| ***Methylotenera mobilis*** | 76 | 0 |
| ***Methylotenera versatilis*** | 71 | 0 |
| ***Methyloversatilis universalis*** | 66 | 0 |
| ***Microbacterium arabinogalactanolyticum*** | 2 | 0 |
| ***Microbacterium aurum*** | 2 | 0 |
| ***Microbacterium chocolatum*** | 6 | 6 |
| ***Microbacterium esteraromaticum*** | 1 | 0 |
| ***Microbacterium flavescens*** | 1 | 1 |
| ***Microbacterium flavum*** | 2 | 0 |
| ***Microbacterium hominis*** | 16 | 0 |
| ***Microbacterium hydrocarbonoxydans*** | 0 | 1 |
| ***Microbacterium ketosireducens*** | 1 | 0 |
| ***Microbacterium koreense*** | 2 | 0 |
| ***Microbacterium lacticum*** | 5 | 0 |
| ***Microbacterium marinilacus*** | 7 | 0 |
| ***Microbacterium maritypicum*** | 1 | 0 |
| ***Microbacterium paludicola*** | 7 | 0 |
| ***Microbacterium pumilum*** | 4 | 0 |
| ***Microbacterium trichothecenolyticum*** | 1 | 0 |
| ***Microbacterium ulmi*** | 0 | 1 |
| ***Microbacterium xinjiangensis*** | 3 | 0 |
| ***Microbacterium xylanilyticum*** | 1 | 0 |
| ***Microbispora rosea*** | 0 | 1 |
| ***Microbulbifer epialgicus*** | 1 | 0 |
| ***Microbulbifer salipaludis*** | 1 | 0 |
| ***Micrococcus luteus*** | 0 | 10 |
| ***Micrococcus yunnanensis*** | 7 | 158 |
| ***Microcoleus antarcticus*** | 1 | 1 |
| ***Microcystis panniformis*** | 1 | 0 |
| ***Microlunatus ginsengisoli*** | 4 | 0 |
| ***Microlunatus phosphovorus*** | 1 | 0 |
| ***Micromonospora purpureochromogenes*** | 0 | 1 |
| ***Micromonospora rifamycinica*** | 1 | 0 |
| ***Microvirus Enterobacteria phage PhiX174*** | 6 | 3 |
| ***Mitsuaria chitosanitabida*** | 0 | 4 |
| ***Mitsuokella jalaludinii*** | 2 | 0 |
| ***Mitsuokella multacida*** | 102 | 0 |
| ***Modestobacter marinus*** | 0 | 3 |
| ***Mogibacterium neglectum*** | 0 | 1 |
| ***Moorella glycerini*** | 0 | 1 |
| ***Moraxella canis*** | 7 | 0 |
| ***Moraxella caviae*** | 46 | 0 |
| ***Moraxella cuniculi*** | 0 | 0 |
| ***Moraxella equi*** | 2 | 0 |
| ***Morganella morganii*** | 1 | 0 |
| ***Mucispirillum schaedleri*** | 0 | 4 |
| ***Muricauda lutimaris*** | 4 | 0 |
| ***Mycetocola lacteus*** | 1 | 0 |
| ***Mycobacterium abscessus*** | 4 | 0 |
| ***Mycobacterium acapulcensis*** | 0 | 0 |
| ***Mycobacterium anthracenicum*** | 11 | 0 |
| ***Mycobacterium arupense*** | 31 | 0 |
| ***Mycobacterium aubagnense*** | 0 | 1 |
| ***Mycobacterium barrassiae*** | 2 | 0 |
| ***Mycobacterium brasiliensis*** | 1 | 0 |
| ***Mycobacterium brisbanense*** | 8 | 0 |
| ***Mycobacterium buckleii*** | 0 | 1 |
| ***Mycobacterium coloregonium*** | 4 | 0 |
| ***Mycobacterium engbaekii*** | 13 | 0 |
| ***Mycobacterium europaeum*** | 1 | 0 |
| ***Mycobacterium fallax*** | 6 | 0 |
| ***Mycobacterium franklinii*** | 5 | 0 |
| ***Mycobacterium frederiksbergense*** | 1 | 0 |
| ***Mycobacterium gordonae*** | 1 | 0 |
| ***Mycobacterium heraklionense*** | 4 | 0 |
| ***Mycobacterium hiberniae*** | 1 | 0 |
| ***Mycobacterium houstonense*** | 0 | 2 |
| ***Mycobacterium insubricum*** | 8 | 0 |
| ***Mycobacterium kansasii*** | 1 | 2 |
| ***Mycobacterium kumamotonense*** | 2 | 0 |
| ***Mycobacterium lacticola*** | 2 | 1 |
| ***Mycobacterium lepromatosis*** | 4 | 0 |
| ***Mycobacterium madagascariense*** | 0 | 3 |
| ***Mycobacterium neglectum*** | 4 | 1 |
| ***Mycobacterium neworleansense*** | 5 | 0 |
| ***Mycobacterium novocastrense*** | 1 | 0 |
| ***Mycobacterium obuense*** | 1 | 0 |
| ***Mycobacterium pallens*** | 11 | 1 |
| ***Mycobacterium parascrofulaceum*** | 4 | 0 |
| ***Mycobacterium pinnipedii*** | 9 | 6 |
| ***Mycobacterium pulveris*** | 1 | 0 |
| ***Mycobacterium pyrenivorans*** | 1 | 0 |
| ***Mycobacterium ratisbonense*** | 41 | 2 |
| ***Mycobacterium rutilum*** | 3 | 4 |
| ***Mycobacterium senuense*** | 1 | 0 |
| ***Mycobacterium simulans*** | 5 | 0 |
| ***Mycobacterium smegmatis*** | 5 | 0 |
| ***Mycobacterium tilburgii*** | 1 | 0 |
| ***Mycobacterium tokaiense*** | 0 | 1 |
| ***Mycobacterium ulcerans*** | 0 | 14 |
| ***Mycobacterium vaccae*** | 1 | 0 |
| ***Mycobacterium vanbaalenii*** | 6 | 0 |
| ***Mycoplasma edwardii*** | 0 | 0 |
| ***Mycoplasma iguanae*** | 4 | 2 |
| ***Mycoplasma insons*** | 7 | 4 |
| ***Mycoplasma timone*** | 6 | 3 |
| ***Myroides odoratus*** | 1 | 0 |
| ***Nannocystis pusilla*** | 2 | 0 |
| ***Natronincola peptidivorans*** | 29 | 18 |
| ***Negativicoccus succinicivorans*** | 3 | 0 |
| ***Neisseria cinerea*** | 0 | 0 |
| ***Neisseria elongata*** | 130 | 3 |
| ***Neisseria flavescens*** | 0 | 0 |
| ***Neisseria lactamica*** | 0 | 5 |
| ***Neisseria mucosa*** | 98 | 58 |
| ***Neisseria subflava*** | 0 | 0 |
| ***Neorickettsia helminthoeca*** | 8 | 0 |
| ***Nesterenkonia flava*** | 0 | 0 |
| ***Nevskia soli*** | 0 | 20 |
| ***Niabella aurantiaca*** | 4 | 0 |
| ***Niabella soli*** | 2 | 0 |
| ***Niastella koreensis*** | 0 | 1 |
| ***Niastella populi*** | 0 | 5 |
| ***Nisaea nitritireducens*** | 0 | 13 |
| ***Nitrincola lacisaponensis*** | 4 | 0 |
| ***Nitrobacter alkalicus*** | 1 | 8 |
| ***Nitrobacter hamburgensis*** | 1 | 35 |
| ***Nitrobacter vulgaris*** | 2 | 336 |
| ***Nitrobacter winogradskyi*** | 0 | 3 |
| ***Nitrosococcus watsoni*** | 3 | 1 |
| ***Nocardia higoensis*** | 0 | 3 |
| ***Nocardia pigrifrangens*** | 0 | 0 |
| ***Nocardia thailandica*** | 2 | 0 |
| ***Nocardioides islandensis*** | 25 | 12 |
| ***Nocardioides lentus*** | 1 | 0 |
| ***Nocardiopsis terrae*** | 1 | 0 |
| ***Nonomuraea asiatica*** | 1 | 0 |
| ***Nostoc ellipsosporum*** | 0 | 0 |
| ***Nostoc entophytum*** | 0 | 117 |
| ***Nostoc microscopicum*** | 0 | 4 |
| ***Nostoc piscinale*** | 0 | 5 |
| ***Novispirillum peregrinum*** | 7 | 0 |
| ***Novosphingobium acidiphilum*** | 2 | 5 |
| ***Novosphingobium aromaticivorans*** | 3 | 1 |
| ***Novosphingobium hassiacum*** | 3 | 101 |
| ***Novosphingobium indicum*** | 8 | 0 |
| ***Novosphingobium mathurense*** | 2 | 1 |
| ***Novosphingobium nitrogenifigens*** | 1 | 0 |
| ***Novosphingobium stygium*** | 0 | 5 |
| ***Novosphingobium taihuense*** | 0 | 0 |
| ***Novosphingobium yangbajingensis*** | 2 | 2 |
| ***Oceanisphaera litoralis*** | 1 | 0 |
| ***Ochrobactrum anthropi*** | 3 | 0 |
| ***Ochrobactrum pecoris*** | 5 | 0 |
| ***Ochrobactrum pseudogrignonense*** | 2 | 0 |
| ***Ochrobactrum thiophenivorans*** | 18 | 1 |
| ***Odoribacter denticanis*** | 2 | 0 |
| ***Odoribacter laneus*** | 1 | 0 |
| ***Oerskovia enterophila*** | 4 | 0 |
| ***Oerskovia ginkgo*** | 1 | 1 |
| ***Oerskovia paurometabola*** | 3 | 0 |
| ***Oleomonas sagaranensis*** | 2 | 4 |
| ***Olivibacter soli*** | 1 | 0 |
| ***Olsenella uli*** | 8 | 0 |
| ***Opitutus terrae*** | 59 | 0 |
| ***Oribacterium sinus*** | 5 | 25 |
| ***Oscillatoria corallinae*** | 0 | 5 |
| ***Oscillospira eae*** | 42 | 6 |
| ***Oscillospira guilliermondii*** | 2 | 0 |
| ***Oxalobacter vibrioformis*** | 217 | 8 |
| ***Paenibacillus caespitis*** | 0 | 12 |
| ***Paenibacillus chitinolyticus*** | 0 | 6 |
| ***Paenibacillus contaminans*** | 0 | 6 |
| ***Paenibacillus dendritiformis*** | 0 | 1 |
| ***Paenibacillus donghaensis*** | 1 | 0 |
| ***Paenibacillus durus*** | 0 | 3 |
| ***Paenibacillus filicis*** | 0 | 13 |
| ***Paenibacillus gansuensis*** | 0 | 1 |
| ***Paenibacillus ginsengihumi*** | 0 | 12 |
| ***Paenibacillus lautus*** | 0 | 5 |
| ***Paenibacillus lentimorbus*** | 0 | 9 |
| ***Paenibacillus mendelii*** | 0 | 4 |
| ***Paenibacillus odorifer*** | 0 | 1 |
| ***Paenibacillus ourofinensis*** | 0 | 1 |
| ***Paenibacillus panacisoli*** | 0 | 2 |
| ***Paenibacillus pini*** | 0 | 1 |
| ***Paenibacillus pinihumi*** | 0 | 4 |
| ***Paenibacillus polymyxa*** | 0 | 2 |
| ***Paenibacillus residui*** | 0 | 5 |
| ***Paenibacillus tarimensis*** | 0 | 7 |
| ***Paenibacillus timonensis*** | 0 | 2 |
| ***Paenibacillus xylanilyticus*** | 0 | 4 |
| ***Paenisporosarcina quisquiliarum*** | 0 | 2 |
| ***Parabacteroides distasonis*** | 36 | 24 |
| ***Parabacteroides goldsteinii*** | 321 | 12 |
| ***Parabacteroides gordonii*** | 0 | 2 |
| ***Parabacteroides johnsonii*** | 3 | 0 |
| ***Parabacteroides merdae*** | 83 | 14 |
| ***Paracoccus aestuarii*** | 4 | 64 |
| ***Paracoccus alkenifer*** | 2 | 6 |
| ***Paracoccus aminophilus*** | 72 | 2 |
| ***Paracoccus aminovorans*** | 108 | 29 |
| ***Paracoccus carotinifaciens*** | 0 | 0 |
| ***Paracoccus chinensis*** | 14 | 6 |
| ***Paracoccus denitrificans*** | 0 | 1 |
| ***Paracoccus haeundaensis*** | 0 | 38 |
| ***Paracoccus halotolerans*** | 1 | 19 |
| ***Paracoccus homiensis*** | 7 | 1524 |
| ***Paracoccus kamogawaensis*** | 0 | 15 |
| ***Paracoccus kocurii*** | 2 | 25 |
| ***Paracoccus koreensis*** | 2 | 0 |
| ***Paracoccus marcusii*** | 75807 | 530 |
| ***Paracoccus marinus*** | 2 | 2 |
| ***Paracoccus sulfuroxidans*** | 101 | 44 |
| ***Paracoccus thiocyanatus*** | 0 | 2 |
| ***Paracoccus yeei*** | 1 | 0 |
| ***Parapedobacter koreensis*** | 287 | 2 |
| ***Paraprevotella clara*** | 1 | 0 |
| ***Pasteurella eae*** | 1 | 0 |
| ***Pectinatus cerevisiiphilus*** | 7 | 80 |
| ***Pectinatus haikarae*** | 6 | 0 |
| ***Pediococcus acidilactici*** | 4 | 0 |
| ***Pediococcus argentinicus*** | 2 | 0 |
| ***Pediococcus cellicola*** | 1 | 0 |
| ***Pedobacter daejeonensis*** | 0 | 0 |
| ***Pedobacter kwangyangensis*** | 184 | 1 |
| ***Pedomicrobium manganicum*** | 1 | 0 |
| ***Pelagicoccus croceus*** | 7 | 0 |
| ***Pelagicoccus mobilis*** | 5 | 0 |
| ***Pelobacter acetylenicus*** | 4 | 0 |
| ***Pelomonas puraquae*** | 1 | 71 |
| ***Pelomonas saccharophila*** | 0 | 9 |
| ***Pelotomaculum isophthalicicum*** | 7 | 7 |
| ***Peptococcus niger*** | 116 | 1 |
| ***Peptoniphilus asaccharolyticus*** | 3 | 30 |
| ***Peptoniphilus coxii*** | 13 | 0 |
| ***Peptoniphilus gorbachii*** | 3 | 10 |
| ***Peptoniphilus indolicus*** | 0 | 0 |
| ***Peptoniphilus ivorii*** | 2 | 2 |
| ***Peptoniphilus lacrimalis*** | 1 | 0 |
| ***Peptoniphilus methioninivorax*** | 363 | 5 |
| ***Peptoniphilus tyrrelliae*** | 0 | 1 |
| ***Peptostreptococcus anaerobius*** | 0 | 4 |
| ***Peptostreptococcus stomatis*** | 0 | 0 |
| ***Phaeobacter inhibens*** | 0 | 1 |
| ***Phaeospirillum fulvum*** | 0 | 3 |
| ***Phascolarctobacterium faecium*** | 18 | 5 |
| ***Phascolarctobacterium succinatutens*** | 97 | 1 |
| ***Phenylobacterium koreense*** | 10 | 3 |
| ***Phenylobacterium lituiforme*** | 0 | 31 |
| ***Phenylobacterium mobile*** | 2 | 3 |
| ***Phormidium murrayi*** | 0 | 1 |
| ***Phycicoccus bigeumensis*** | 2 | 0 |
| ***Phycicoccus dokdonensis*** | 1 | 1 |
| ***Phycicoccus jejuensis*** | 3 | 0 |
| ***Phyllobacterium bourgognense*** | 4 | 0 |
| ***Phyllobacterium brassicacearum*** | 0 | 1 |
| ***Phyllobacterium catacumbae*** | 2 | 4 |
| ***Pimelobacter simplex*** | 1 | 3 |
| ***Pirellula staleyi*** | 1 | 2 |
| ***Planctomyces limnophilus*** | 8 | 0 |
| ***Planctomyces maris*** | 1 | 0 |
| ***Planifilum fimeticola*** | 122 | 0 |
| ***Planococcus maritimus*** | 8 | 11 |
| ***Planococcus rifietoensis*** | 0 | 0 |
| ***Planomicrobium flavidum*** | 0 | 2 |
| ***Planomicrobium glaciei*** | 0 | 0 |
| ***Planomicrobium okeanokoites*** | 0 | 2 |
| ***Pleomorphomonas koreensis*** | 57 | 0 |
| ***Pleomorphomonas oryzae*** | 1 | 0 |
| ***Polaribacter butkevichii*** | 8 | 3 |
| ***Polaromonas jejuensis*** | 0 | 5 |
| ***Polaromonas naphthalenivorans*** | 4 | 0 |
| ***Pontibacillus halophilus*** | 0 | 0 |
| ***Pontibacter niistensis*** | 0 | 5 |
| ***Porphyromonas cangingivalis*** | 0 | 5 |
| ***Porphyromonas canis*** | 43 | 2 |
| ***Porphyromonas canoris*** | 0 | 0 |
| ***Porphyromonas catoniae*** | 13 | 12 |
| ***Porphyromonas circumdentaria*** | 40 | 1 |
| ***Porphyromonas endodontalis*** | 0 | 13 |
| ***Porphyromonas somerae*** | 0 | 7 |
| ***Prevotella albensis*** | 1 | 1 |
| ***Prevotella amnii*** | 0 | 2 |
| ***Prevotella aurantiaca*** | 0 | 0 |
| ***Prevotella bergensis*** | 0 | 1 |
| ***Prevotella bivia*** | 0 | 66 |
| ***Prevotella buccalis*** | 1 | 0 |
| ***Prevotella copri*** | 43 | 19 |
| ***Prevotella corporis*** | 0 | 8 |
| ***Prevotella dentalis*** | 1 | 0 |
| ***Prevotella disiens*** | 0 | 12 |
| ***Prevotella histicola*** | 0 | 40 |
| ***Prevotella intermedia*** | 0 | 1 |
| ***Prevotella loescheii*** | 0 | 0 |
| ***Prevotella melaninogenica*** | 0 | 412 |
| ***Prevotella multiformis*** | 0 | 15 |
| ***Prevotella multisaccharivorax*** | 3 | 0 |
| ***Prevotella nanceiensis*** | 0 | 15 |
| ***Prevotella nigrescens*** | 0 | 5 |
| ***Prevotella oris*** | 0 | 11 |
| ***Prevotella oulorum*** | 0 | 2 |
| ***Prevotella pallens*** | 0 | 18 |
| ***Prevotella paludivivens*** | 4 | 0 |
| ***Prevotella salivae*** | 0 | 9 |
| ***Prevotella shahii*** | 1 | 0 |
| ***Prevotella stercorea*** | 1 | 0 |
| ***Prevotella tannerae*** | 0 | 0 |
| ***Prevotella timonensis*** | 1 | 185 |
| ***Prevotella veroralis*** | 0 | 3 |
| ***Prochlorococcus marinus*** | 3 | 0 |
| ***Promicromonospora kroppenstedtii*** | 0 | 0 |
| ***Promicromonospora umidemergens*** | 0 | 1 |
| ***Propionibacterium acidipropionici*** | 1 | 0 |
| ***Propionibacterium acnes*** | 8 | 84 |
| ***Propionibacterium avidum*** | 37 | 5 |
| ***Propionibacterium humerusii*** | 5 | 20 |
| ***Propionibacterium microaerophilum*** | 21 | 3 |
| ***Propionibacterium thoenii*** | 6 | 0 |
| ***Propionicimonas paludicola*** | 87 | 0 |
| ***Propionigenium modestum*** | 774 | 0 |
| ***Propionispora hippei*** | 1039 | 9 |
| ***Propionivibrio dicarboxylicus*** | 63 | 19 |
| ***Propionivibrio limicola*** | 71 | 0 |
| ***Propionivibrio pelophilus*** | 121 | 0 |
| ***Prosthecobacter debontii*** | 12 | 0 |
| ***Prosthecobacter dejongeii*** | 2 | 0 |
| ***Proteus hauseri*** | 0 | 1 |
| ***Proteus myxofaciens*** | 0 | 5 |
| ***Proteus penneri*** | 0 | 3 |
| ***Pseudaminobacter defluvii*** | 3 | 0 |
| ***Pseudidiomarina donghaiensis*** | 0 | 0 |
| ***Pseudobutyrivibrio xylanivorans*** | 3 | 18 |
| ***Pseudochrobactrum kiredjianiae*** | 2 | 0 |
| ***Pseudochrobactrum saccharolyticum*** | 2 | 0 |
| ***Pseudoclavibacter bifida*** | 2 | 0 |
| ***Pseudomonas aeruginosa*** | 2 | 7 |
| ***Pseudomonas agarici*** | 8 | 0 |
| ***Pseudomonas alcaligenes*** | 63 | 0 |
| ***Pseudomonas alkylphenolia*** | 7 | 2 |
| ***Pseudomonas amygdali*** | 3 | 0 |
| ***Pseudomonas anguilliseptica*** | 59 | 0 |
| ***Pseudomonas azotoformans*** | 228 | 0 |
| ***Pseudomonas benzenivorans*** | 459 | 1 |
| ***Pseudomonas borealis*** | 2 | 0 |
| ***Pseudomonas brassicacearum*** | 9 | 0 |
| ***Pseudomonas brenneri*** | 667 | 0 |
| ***Pseudomonas caricapapayae*** | 1 | 0 |
| ***Pseudomonas chloritidismutans*** | 4 | 0 |
| ***Pseudomonas cichorii*** | 7 | 0 |
| ***Pseudomonas cinnamophila*** | 8 | 0 |
| ***Pseudomonas clemancea*** | 41 | 0 |
| ***Pseudomonas collierea*** | 17 | 0 |
| ***Pseudomonas coronafaciens*** | 128 | 0 |
| ***Pseudomonas corrugata*** | 85 | 0 |
| ***Pseudomonas cremoricolorata*** | 749 | 1 |
| ***Pseudomonas entomophila*** | 17 | 1 |
| ***Pseudomonas fluorescens*** | 227 | 0 |
| ***Pseudomonas fragi*** | 6919 | 17 |
| ***Pseudomonas fulva*** | 1 | 0 |
| ***Pseudomonas fuscovaginae*** | 4 | 0 |
| ***Pseudomonas gessardii*** | 9 | 0 |
| ***Pseudomonas gingeri*** | 2 | 0 |
| ***Pseudomonas guineae*** | 38 | 0 |
| ***Pseudomonas japonica*** | 0 | 1 |
| ***Pseudomonas jessenii*** | 8 | 0 |
| ***Pseudomonas jinjuensis*** | 2 | 0 |
| ***Pseudomonas koreensis*** | 1 | 0 |
| ***Pseudomonas lini*** | 20 | 0 |
| ***Pseudomonas lundensis*** | 10748 | 2 |
| ***Pseudomonas lurida*** | 10 | 0 |
| ***Pseudomonas mandelii*** | 501 | 0 |
| ***Pseudomonas marginalis*** | 6074 | 3 |
| ***Pseudomonas mediterranea*** | 2 | 0 |
| ***Pseudomonas meliae*** | 20 | 0 |
| ***Pseudomonas mendocina*** | 2 | 6 |
| ***Pseudomonas metavorans*** | 29 | 0 |
| ***Pseudomonas migulae*** | 20 | 0 |
| ***Pseudomonas moorei*** | 1 | 0 |
| ***Pseudomonas moraviensis*** | 136 | 1 |
| ***Pseudomonas mosselii*** | 169 | 0 |
| ***Pseudomonas mucidolens*** | 5 | 0 |
| ***Pseudomonas oryzihabitans*** | 9 | 0 |
| ***Pseudomonas otitidis*** | 2 | 5 |
| ***Pseudomonas panacis*** | 4 | 0 |
| ***Pseudomonas panipatensis*** | 49 | 0 |
| ***Pseudomonas parafulva*** | 51 | 4 |
| ***Pseudomonas pavonaceae*** | 1 | 0 |
| ***Pseudomonas plecoglossicida*** | 10228 | 21 |
| ***Pseudomonas poae*** | 29 | 0 |
| ***Pseudomonas proteolytica*** | 560 | 1 |
| ***Pseudomonas pseudoalcaligenes*** | 204 | 0 |
| ***Pseudomonas psychrophila*** | 4 | 0 |
| ***Pseudomonas putida*** | 146 | 0 |
| ***Pseudomonas reinekei*** | 3 | 0 |
| ***Pseudomonas resinovorans*** | 7 | 0 |
| ***Pseudomonas rhodesiae*** | 14 | 0 |
| ***Pseudomonas savastanoi*** | 139 | 0 |
| ***Pseudomonas stutzeri*** | 0 | 14 |
| ***Pseudomonas syncyanea*** | 76 | 0 |
| ***Pseudomonas syringae*** | 28 | 0 |
| ***Pseudomonas taiwanensis*** | 3 | 0 |
| ***Pseudomonas teessidea*** | 44 | 0 |
| ***Pseudomonas thermotolerans*** | 2 | 0 |
| ***Pseudomonas tolaasii*** | 6 | 0 |
| ***Pseudomonas tremae*** | 1353 | 4 |
| ***Pseudomonas trivialis*** | 7 | 0 |
| ***Pseudomonas tropicalis*** | 0 | 2 |
| ***Pseudomonas umsongensis*** | 102 | 1 |
| ***Pseudomonas vancouverensis*** | 197 | 0 |
| ***Pseudomonas veronii*** | 1055 | 0 |
| ***Pseudomonas viridiflava*** | 10 | 5 |
| ***Pseudomonas xanthomarina*** | 5 | 0 |
| ***Pseudonocardia acaciae*** | 1 | 0 |
| ***Pseudonocardia asaccharolytica*** | 1 | 1 |
| ***Pseudonocardia babensis*** | 1 | 1 |
| ***Pseudonocardia compacta*** | 3 | 0 |
| ***Pseudonocardia hydrocarbonoxydans*** | 0 | 6 |
| ***Pseudonocardia khuvsgulensis*** | 0 | 1 |
| ***Pseudonocardia kongjuensis*** | 3 | 4 |
| ***Pseudonocardia oroxyli*** | 1 | 0 |
| ***Pseudonocardia tetrahydrofuranoxydans*** | 0 | 2 |
| ***Pseudoramibacter alactolyticus*** | 96 | 0 |
| ***Pseudoxanthomonas indica*** | 1 | 0 |
| ***Pseudoxanthomonas japonensis*** | 13 | 0 |
| ***Pseudoxanthomonas mexicana*** | 65 | 0 |
| ***Pseudoxanthomonas sacheonensis*** | 2 | 1 |
| ***Pseudoxanthomonas yeongjuensis*** | 8 | 0 |
| ***Psychrobacter cibarius*** | 33 | 0 |
| ***Psychrobacter cryohalolentis*** | 1 | 0 |
| ***Psychrobacter glacialis*** | 3 | 0 |
| ***Psychrobacter glacincola*** | 13 | 0 |
| ***Psychrobacter halophilus*** | 1 | 1 |
| ***Psychrobacter phenylpyruvicus*** | 1 | 0 |
| ***Psychrobacter proteolyticus*** | 0 | 4 |
| ***Psychrobacter pulmonis*** | 1 | 1 |
| ***Psychroflexus gondwanensis*** | 38 | 0 |
| ***Psychromonas aquimarina*** | 8 | 0 |
| ***Psychroserpens burtonensis*** | 1 | 0 |
| ***Pullulanibacillus naganoensis*** | 0 | 7 |
| ***Pyramidobacter piscolens*** | 1 | 0 |
| ***Ralstonia detusculanense*** | 4 | 92 |
| ***Ralstonia insidiosa*** | 1 | 31 |
| ***Ralstonia mannitolilytica*** | 1 | 0 |
| ***Ralstonia pickettii*** | 0 | 20 |
| ***Ramlibacter tataouinensis*** | 7 | 0 |
| ***Rheinheimera chironomi*** | 12 | 0 |
| ***Rheinheimera soli*** | 66 | 0 |
| ***Rheinheimera texasensis*** | 2 | 0 |
| ***Rhizobium alamii*** | 75 | 0 |
| ***Rhizobium indigoferae*** | 1 | 0 |
| ***Rhodobaca bogoriensis*** | 1 | 0 |
| ***Rhodobacter blasticus*** | 2 | 0 |
| ***Rhodobacter gluconicum*** | 17 | 6 |
| ***Rhodobacter ovatus*** | 4 | 21 |
| ***Rhodococcus baikonurensis*** | 1 | 0 |
| ***Rhodococcus equi*** | 2 | 0 |
| ***Rhodococcus imtechensis*** | 1 | 0 |
| ***Rhodococcus percolatus*** | 2 | 1 |
| ***Rhodococcus phenolicus*** | 14 | 0 |
| ***Rhodococcus pyridinivorans*** | 1 | 0 |
| ***Rhodococcus qingshengii*** | 4 | 0 |
| ***Rhodocyclus purpureus*** | 20 | 1 |
| ***Rhodoferax antarcticus*** | 1 | 0 |
| ***Rhodoferax ferrireducens*** | 1 | 0 |
| ***Rhodothermus clarus*** | 15 | 1 |
| ***Rhodovibrio sodomensis*** | 6 | 7 |
| ***Rhodovulum euryhalinum*** | 2 | 0 |
| ***Rickettsia australis*** | 0 | 3 |
| ***Rickettsia marmionii*** | 1 | 0 |
| ***Rickettsia monacensis*** | 5 | 2 |
| ***Rikenella microfusus*** | 90 | 0 |
| ***Roseburia faecis*** | 12 | 22 |
| ***Roseivivax halodurans*** | 0 | 2 |
| ***Roseococcus thiosulfatophilus*** | 0 | 1 |
| ***Roseomonas aquatica*** | 0 | 6 |
| ***Roseomonas lacus*** | 2 | 3 |
| ***Roseomonas massiliensis*** | 3 | 0 |
| ***Roseomonas mucosa*** | 1 | 10 |
| ***Roseomonas terpenica*** | 2 | 5 |
| ***Roseomonas terrae*** | 6 | 1 |
| ***Roseospira thiosulfatophila*** | 0 | 0 |
| ***Roseospira visakhapatnamensis*** | 2 | 0 |
| ***Rothia aeria*** | 0 | 15 |
| ***Rothia dentocariosa*** | 1 | 114 |
| ***Rothia mucilaginosa*** | 2 | 202 |
| ***Rubellimicrobium aerolatum*** | 1 | 30 |
| ***Rubellimicrobium mesophilum*** | 0 | 2 |
| ***Rubellimicrobium roseum*** | 0 | 2 |
| ***Rubellimicrobium thermophilum*** | 0 | 1 |
| ***Rubrivivax gelatinosus*** | 2 | 256 |
| ***Rubrobacter taiwanensis*** | 1 | 0 |
| ***Ruegeria lacuscaerulensis*** | 9 | 156 |
| ***Ruegeria pomeroyi*** | 1 | 0 |
| ***Ruminococcus albus*** | 2 | 2 |
| ***Ruminococcus bromii*** | 141 | 20 |
| ***Ruminococcus callidus*** | 5 | 6 |
| ***Ruminococcus gnavus*** | 27 | 107 |
| ***Ruminococcus torques*** | 9 | 8 |
| ***Rummeliibacillus pycnus*** | 0 | 3 |
| ***Rummeliibacillus stabekisii*** | 1 | 0 |
| ***Runella limosa*** | 14 | 7 |
| ***Saccharopolyspora cebuensis*** | 1 | 0 |
| ***Saccharopolyspora shandongensis*** | 7 | 0 |
| ***Salinibacterium xinjiangense*** | 1 | 4 |
| ***Salinicoccus luteus*** | 0 | 0 |
| ***Salinimicrobium terrae*** | 1 | 0 |
| ***Salinispora tropica*** | 3 | 0 |
| ***Salinivibrio budaii*** | 2 | 0 |
| ***Salmonella enterica*** | 1 | 0 |
| ***Sanguibacter suarezii*** | 56 | 0 |
| ***Sarcina maxima*** | 57 | 0 |
| ***Sarcina ventriculi*** | 19 | 0 |
| ***Scardovia wiggsiae*** | 1 | 0 |
| ***Schlegelella aquatica*** | 1 | 2 |
| ***Sebaldella termitidis*** | 91 | 0 |
| ***Sedimentibacter hydroxybenzoicus*** | 248 | 1 |
| ***Segetibacter aerophilus*** | 16 | 0 |
| ***Segetibacter koreensis*** | 0 | 2 |
| ***Selenomonas artemidis*** | 1 | 0 |
| ***Selenomonas infelix*** | 13 | 1 |
| ***Serinicoccus chungangensis*** | 2 | 1 |
| ***Serratia entomophila*** | 28 | 162 |
| ***Serratia marcescens*** | 0 | 8 |
| ***Sharpea azabuensis*** | 5 | 3 |
| ***Shewanella decolorationis*** | 3 | 0 |
| ***Shewanella gaetbuli*** | 2 | 0 |
| ***Shewanella livingstonensis*** | 9 | 0 |
| ***Shewanella oneidensis*** | 387 | 0 |
| ***Shewanella pneumatophori*** | 2 | 0 |
| ***Shewanella profunda*** | 1853 | 0 |
| ***Shewanella putrefaciens*** | 4421 | 1 |
| ***Shewanella upenei*** | 48 | 1 |
| ***Shinella fusca*** | 10 | 8 |
| ***Shinella granuli*** | 150 | 0 |
| ***Shinella yambaruensis*** | 3 | 0 |
| ***Sinorhizobium fredii*** | 0 | 1 |
| ***Skermanella aerolata*** | 3 | 2 |
| ***Slackia faecicanis*** | 1 | 1 |
| ***Slackia isoflavoniconvertens*** | 32 | 0 |
| ***Slackia piriformis*** | 20 | 1 |
| ***Sneathia sanguinegens*** | 46 | 0 |
| ***Snowella rosea*** | 5 | 3 |
| ***Soehngenia saccharolytica*** | 13 | 0 |
| ***Solirubrobacter soli*** | 1 | 4 |
| ***Sphaerochaeta coccoides*** | 1 | 0 |
| ***Sphaerochaeta globus*** | 1 | 0 |
| ***Sphingobacterium bambusae*** | 3214 | 0 |
| ***Sphingobacterium composti*** | 1 | 4 |
| ***Sphingobacterium mizutaii*** | 1 | 0 |
| ***Sphingobacterium shayense*** | 257 | 5 |
| ***Sphingobium abikonense*** | 0 | 1 |
| ***Sphingobium amiense*** | 4 | 17 |
| ***Sphingobium faniae*** | 1 | 2 |
| ***Sphingobium olei*** | 0 | 6 |
| ***Sphingobium rhizovicinum*** | 0 | 1 |
| ***Sphingobium ummariense*** | 1 | 2 |
| ***Sphingobium yanoikuyae*** | 6841 | 153 |
| ***Sphingomonas abaci*** | 1 | 1 |
| ***Sphingomonas asaccharolytica*** | 1 | 1 |
| ***Sphingomonas azotifigens*** | 0 | 0 |
| ***Sphingomonas dokdonensis*** | 2 | 43 |
| ***Sphingomonas elodea*** | 0 | 1 |
| ***Sphingomonas ginsenosidimutans*** | 1 | 4 |
| ***Sphingomonas haloaromaticamans*** | 1 | 0 |
| ***Sphingomonas hankookensis*** | 0 | 0 |
| ***Sphingomonas hunanensis*** | 0 | 1 |
| ***Sphingomonas insulae*** | 0 | 120 |
| ***Sphingomonas japonica*** | 0 | 0 |
| ***Sphingomonas mali*** | 0 | 0 |
| ***Sphingomonas melonis*** | 0 | 11 |
| ***Sphingomonas oligophenolica*** | 12 | 291 |
| ***Sphingomonas panni*** | 2 | 95 |
| ***Sphingomonas roseiflava*** | 0 | 0 |
| ***Sphingomonas sanxanigenens*** | 0 | 4 |
| ***Sphingomonas soli*** | 0 | 1 |
| ***Sphingomonas wittichii*** | 5 | 4 |
| ***Sphingomonas yabuuchiae*** | 0 | 0 |
| ***Sphingomonas yunnanensis*** | 0 | 1 |
| ***Sphingopyxis alaskensis*** | 1 | 0 |
| ***Sphingopyxis granuli*** | 1 | 0 |
| ***Sphingopyxis taejonensis*** | 20 | 0 |
| ***Sphingopyxis terrae*** | 17 | 0 |
| ***Sphingopyxis witflariensis*** | 5 | 1 |
| ***Spirosoma linguale*** | 0 | 1 |
| ***Sporolactobacillus putidus*** | 0 | 0 |
| ***Sporosarcina contaminans*** | 0 | 1 |
| ***Sporosarcina pasteurii*** | 4 | 0 |
| ***Sporosarcina soli*** | 0 | 7 |
| ***Sporotomaculum syntrophicum*** | 3 | 19 |
| ***Staphylococcus aureus*** | 3 | 207 |
| ***Staphylococcus auricularis*** | 1 | 0 |
| ***Staphylococcus capitis*** | 0 | 1 |
| ***Staphylococcus caprae*** | 1 | 11 |
| ***Staphylococcus cohnii*** | 1 | 0 |
| ***Staphylococcus epidermidis*** | 1 | 3 |
| ***Staphylococcus gallinarum*** | 0 | 1 |
| ***Staphylococcus haemolyticus*** | 0 | 1 |
| ***Staphylococcus hominis*** | 0 | 13 |
| ***Staphylococcus lugdunensis*** | 1 | 50 |
| ***Staphylococcus pasteuri*** | 0 | 1 |
| ***Staphylococcus pseudolugdunensis*** | 0 | 2 |
| ***Staphylococcus warneri*** | 0 | 1 |
| ***Stenotrophomonas acidaminiphila*** | 34 | 0 |
| ***Stenotrophomonas chelatiphaga*** | 0 | 2 |
| ***Stenotrophomonas geniculata*** | 4 | 92 |
| ***Stenotrophomonas koreensis*** | 25 | 0 |
| ***Stenotrophomonas maltophilia*** | 1 | 2 |
| ***Stenotrophomonas nitritireducens*** | 2 | 0 |
| ***Stenotrophomonas pavanii*** | 3 | 23 |
| ***Stenotrophomonas retroflexus*** | 1 | 0 |
| ***Stenotrophomonas terrae*** | 10 | 0 |
| ***Steroidobacter denitrificans*** | 1 | 1 |
| ***Streptobacillus moniliformis*** | 35 | 0 |
| ***Streptococcus agalactiae*** | 1 | 0 |
| ***Streptococcus alactolyticus*** | 10 | 0 |
| ***Streptococcus anginosus*** | 4 | 3 |
| ***Streptococcus australis*** | 6 | 19 |
| ***Streptococcus bovis*** | 1315 | 34 |
| ***Streptococcus canis*** | 0 | 6 |
| ***Streptococcus castoreus*** | 10 | 0 |
| ***Streptococcus cristatus*** | 0 | 30 |
| ***Streptococcus dentapri*** | 1 | 0 |
| ***Streptococcus dentirousetti*** | 0 | 3 |
| ***Streptococcus equinus*** | 1266 | 0 |
| ***Streptococcus ferus*** | 21 | 0 |
| ***Streptococcus fryi*** | 112 | 7 |
| ***Streptococcus gallinaceus*** | 61 | 0 |
| ***Streptococcus gordonii*** | 2 | 94 |
| ***Streptococcus infantarius*** | 51 | 6 |
| ***Streptococcus infantis*** | 2 | 69 |
| ***Streptococcus intermedius*** | 3 | 0 |
| ***Streptococcus lactarius*** | 7 | 1 |
| ***Streptococcus marimammalium*** | 1 | 0 |
| ***Streptococcus milleri*** | 10 | 11 |
| ***Streptococcus minor*** | 9144 | 4 |
| ***Streptococcus mutans*** | 12 | 0 |
| ***Streptococcus oligofermentans*** | 0 | 2 |
| ***Streptococcus oralis*** | 4 | 101 |
| ***Streptococcus orisratti*** | 53 | 0 |
| ***Streptococcus parasanguinis*** | 31 | 56 |
| ***Streptococcus peroris*** | 3 | 15 |
| ***Streptococcus phocae*** | 0 | 4 |
| ***Streptococcus plurextorum*** | 3 | 0 |
| ***Streptococcus pseudopneumoniae*** | 4 | 79 |
| ***Streptococcus sanguinis*** | 1 | 35 |
| ***Streptococcus thermophilus*** | 86 | 20 |
| ***Streptococcus tigurinus*** | 1 | 143 |
| ***Streptococcus troglodytae*** | 2 | 0 |
| ***Streptococcus ursoris*** | 7 | 0 |
| ***Streptococcus vestibularis*** | 396 | 205 |
| ***Streptomyces alni*** | 0 | 2 |
| ***Streptomyces aureoversilis*** | 0 | 1 |
| ***Streptomyces danangensis*** | 1 | 0 |
| ***Streptomyces eurythermus*** | 0 | 1 |
| ***Streptomyces labedae*** | 0 | 3 |
| ***Streptomyces lazureus*** | 1 | 0 |
| ***Streptomyces libani*** | 0 | 1 |
| ***Streptomyces mycarofaciens*** | 0 | 1 |
| ***Streptomyces pulcher*** | 2 | 0 |
| ***Streptomyces radiopugnans*** | 0 | 1 |
| ***Streptomyces roseogilvus*** | 3 | 0 |
| ***Streptomyces violens*** | 0 | 13 |
| ***Succiniclasticum ruminis*** | 1 | 0 |
| ***Sulfobacillus yellowstonensis*** | 0 | 3 |
| ***Sulfuricurvum kujiense*** | 847 | 0 |
| ***Sulfurimonas paralvinellae*** | 1 | 0 |
| ***Sulfurospirillum arcachonense*** | 1 | 0 |
| ***Sulfurospirillum deleyianum*** | 11 | 0 |
| ***Sutterella sanguinus*** | 2 | 0 |
| ***Sutterella stercoricanis*** | 0 | 1 |
| ***Sutterella wadsworthensis*** | 0 | 8 |
| ***Symploca atlantica*** | 2 | 0 |
| ***Syntrophobacter sulfatireducens*** | 1 | 0 |
| ***Syntrophobacter wolinii*** | 13 | 0 |
| ***Syntrophomonas cellicola*** | 1 | 6 |
| ***Syntrophomonas sapovorans*** | 0 | 1 |
| ***Telmatospirillum siberiense*** | 3 | 20 |
| ***Tenacibaculum litopenaei*** | 1 | 0 |
| ***Tenacibaculum litoreum*** | 1 | 0 |
| ***Tepidanaerobacter syntrophicus*** | 2 | 0 |
| ***Tepidimonas ignava*** | 6 | 1 |
| ***Tepidimonas taiwanensis*** | 1 | 0 |
| ***Tepidimonas thermarum*** | 4 | 0 |
| ***Tetragenococcus doogicus*** | 1 | 2 |
| ***Tetrasphaera australiensis*** | 5 | 0 |
| ***Tetrasphaera jenkinsii*** | 1 | 0 |
| ***Tetrasphaera vanveenii*** | 4 | 0 |
| ***Thalassospira tepidiphila*** | 23 | 2 |
| ***Thalassospira xianhensis*** | 9 | 0 |
| ***Thauera aromatica*** | 127 | 0 |
| ***Thauera linaloolentis*** | 9 | 1 |
| ***Thauera mechernichensis*** | 188 | 2 |
| ***Thauera selenatis*** | 177 | 0 |
| ***Thauera terpenica*** | 48 | 0 |
| ***Thermicanus aegyptius*** | 0 | 2 |
| ***Thermoactinomyces intermedius*** | 0 | 1 |
| ***Thermoactinomyces vulgaris*** | 0 | 5 |
| ***Thermoanaerobacter inferii*** | 0 | 4 |
| ***Thermoanaerobacter kivui*** | 0 | 1 |
| ***Thermoanaerobacterium islandicum*** | 2 | 1 |
| ***Thermobaculum terrenum*** | 9 | 0 |
| ***Thermodesulfovibrio aggregans*** | 4 | 0 |
| ***Thermodesulfovibrio thiophilus*** | 12 | 0 |
| ***Thermogemmatispora onikobensis*** | 3 | 0 |
| ***Thermomonas brevis*** | 5 | 0 |
| ***Thermomonas dokdonensis*** | 3 | 0 |
| ***Thermomonas fusca*** | 14 | 0 |
| ***Thermomonas haemolytica*** | 7 | 0 |
| ***Thermosipho ferriphilus*** | 0 | 2 |
| ***Thermovenabulum ferriorganovorum*** | 23 | 4 |
| ***Thermovirga lienii*** | 1 | 0 |
| ***Thermus scotoductus*** | 0 | 3 |
| ***Thermus thermophilus*** | 0 | 4 |
| ***Thioalkalimicrobium sibiricum*** | 2 | 1 |
| ***Thioalkalivibrio jannaschii*** | 1 | 0 |
| ***Thiobacillus sajanensis*** | 9 | 4 |
| ***Thiobacillus thioparus*** | 1 | 0 |
| ***Thiohalorhabdus denitrificans*** | 74 | 1 |
| ***Thiomonas intermedia*** | 13 | 0 |
| ***Thiomonas perometabolis*** | 5 | 0 |
| ***Thiomonas thermosulfata*** | 6 | 180 |
| ***Thiorhodococcus mannitoliphagus*** | 0 | 4 |
| ***Thiorhodococcus pfennigii*** | 0 | 2 |
| ***Thiothrix caldifontis*** | 1 | 0 |
| ***Thiothrix eikelboomii*** | 2 | 0 |
| ***Thiothrix lacustris*** | 8 | 0 |
| ***Thiovirga sulfuroxydans*** | 12 | 0 |
| ***Tindallia magadiensis*** | 6 | 3 |
| ***Tolumonas auensis*** | 996 | 11 |
| ***Trabulsiella farmeri*** | 29 | 165 |
| ***Trabulsiella guamensis*** | 0 | 7 |
| ***Trabulsiella odontotermitis*** | 1 | 4 |
| ***Treponema brennaborense*** | 11 | 0 |
| ***Treponema bryantii*** | 2 | 0 |
| ***Treponema maltophilum*** | 0 | 1 |
| ***Treponema paraluiscuniculi*** | 2 | 0 |
| ***Treponema porcinum*** | 5 | 0 |
| ***Treponema succinifaciens*** | 1 | 0 |
| ***Treponema zioleckii*** | 8 | 0 |
| ***Trichodesmium havanum*** | 0 | 1 |
| ***Trichodesmium tenue*** | 0 | 1 |
| ***Turicibacter sanguinis*** | 30 | 1 |
| ***Uliginosibacterium gangwonense*** | 8 | 5 |
| ***Ureaplasma gallorale*** | 0 | 1 |
| ***Ureaplasma parvum*** | 0 | 12 |
| ***Ureibacillus thermophilus*** | 1 | 0 |
| ***Vagococcus fluvialis*** | 1 | 0 |
| ***Vagococcus penaei*** | 1 | 0 |
| ***Vagococcus teuberi*** | 10 | 4 |
| ***Varibaculum cambriense*** | 0 | 4 |
| ***Variovorax boronicumulans*** | 26 | 4 |
| ***Variovorax dokdonensis*** | 1 | 0 |
| ***Variovorax paradoxus*** | 1 | 109 |
| ***Variovorax soli*** | 20 | 2 |
| ***Veillonella atypica*** | 3 | 36 |
| ***Veillonella criceti*** | 1 | 0 |
| ***Veillonella denticariosi*** | 0 | 2 |
| ***Veillonella dispar*** | 3 | 28 |
| ***Veillonella montpellierensis*** | 182 | 3 |
| ***Veillonella parvula*** | 5 | 9 |
| ***Veillonella ratti*** | 2 | 0 |
| ***Verrucosispora gifhornensis*** | 0 | 9 |
| ***Vibrio litoralis*** | 0 | 1 |
| ***Virgibacillus byunsanensis*** | 0 | 1 |
| ***Virgibacillus picturae*** | 0 | 18 |
| ***Virgibacillus salexigens*** | 2 | 5 |
| ***Virgisporangium aurantiacum*** | 0 | 1 |
| ***Viridibacillus neidei*** | 5 | 0 |
| ***Vitreoscilla stercoraria*** | 1 | 0 |
| ***Vogesella perlucida*** | 18 | 4 |
| ***Weissella cibaria*** | 3 | 6 |
| ***Winogradskyella exilis*** | 3 | 0 |
| ***Winogradskyella rapida*** | 1 | 0 |
| ***Xanthobacter polyaromaticivorans*** | 18 | 8 |
| ***Xanthobacter tagetidis*** | 1 | 0 |
| ***Xanthobacter viscosus*** | 0 | 1 |
| ***Xanthomonas albilineans*** | 1 | 0 |
| ***Xanthomonas oryzae*** | 20 | 0 |
| ***Xanthomonas sacchari*** | 1 | 0 |
| ***Yersinia enterocolitica*** | 5 | 0 |
| ***Yersinia frederiksenii*** | 2 | 0 |
| ***Yersinia intermedia*** | 1 | 0 |
| ***Yersinia kristensenii*** | 1 | 0 |
| ***Yersinia massiliensis*** | 1 | 0 |
| ***Yonghaparkia alkaliphila*** | 0 | 26 |
| ***Zhouia amylolytica*** | 2 | 0 |
| ***Zobellia laminariae*** | 20 | 0 |
| ***Zoogloea caeni*** | 6 | 0 |
| ***Zoogloea oryzae*** | 24 | 0 |
| ***Zoogloea ramigera*** | 80 | 0 |
| ***Zoogloea resiniphila*** | 8 | 0 |

**Supplementary figure 2**


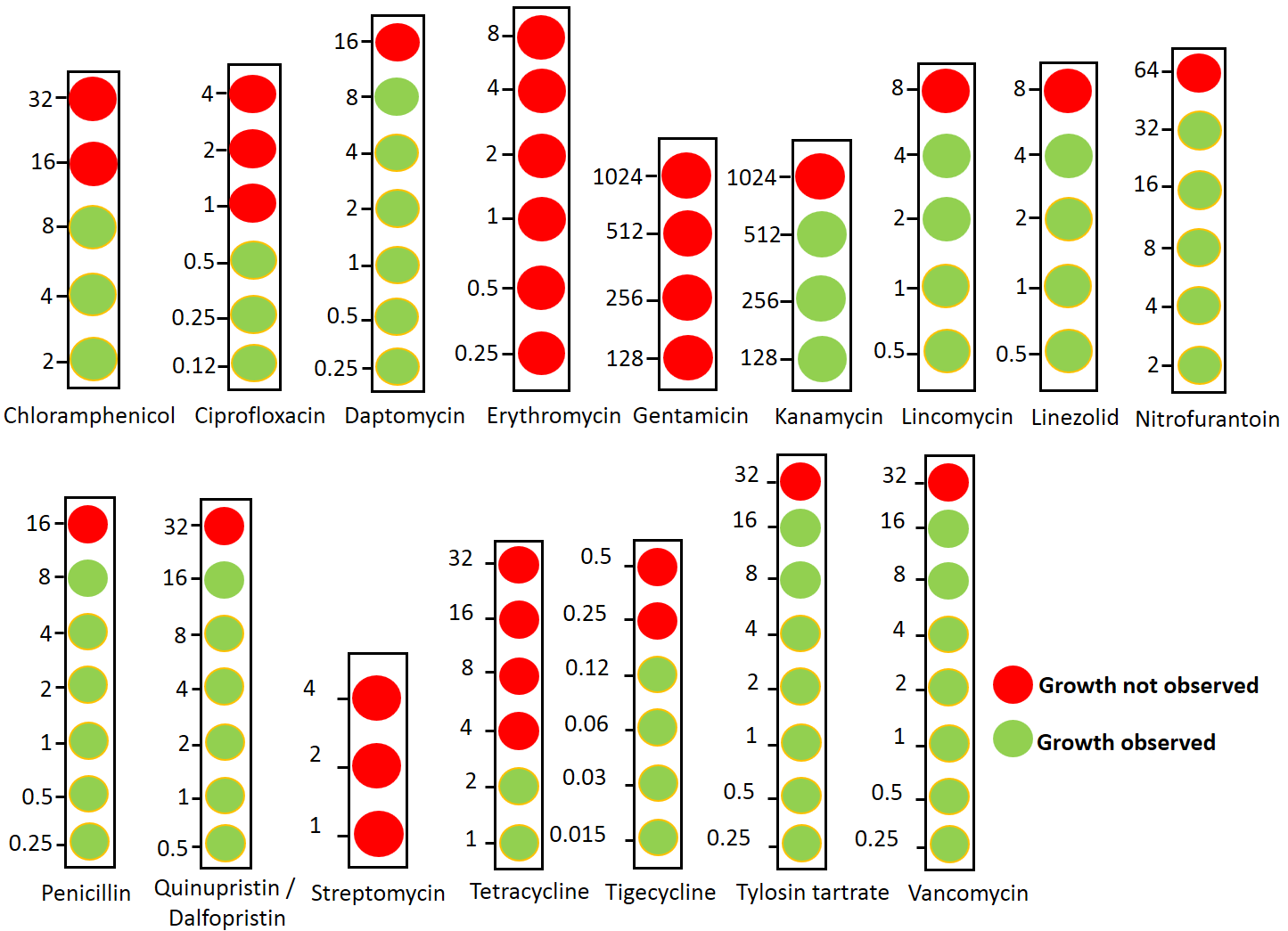


**Supplementary figure 3**


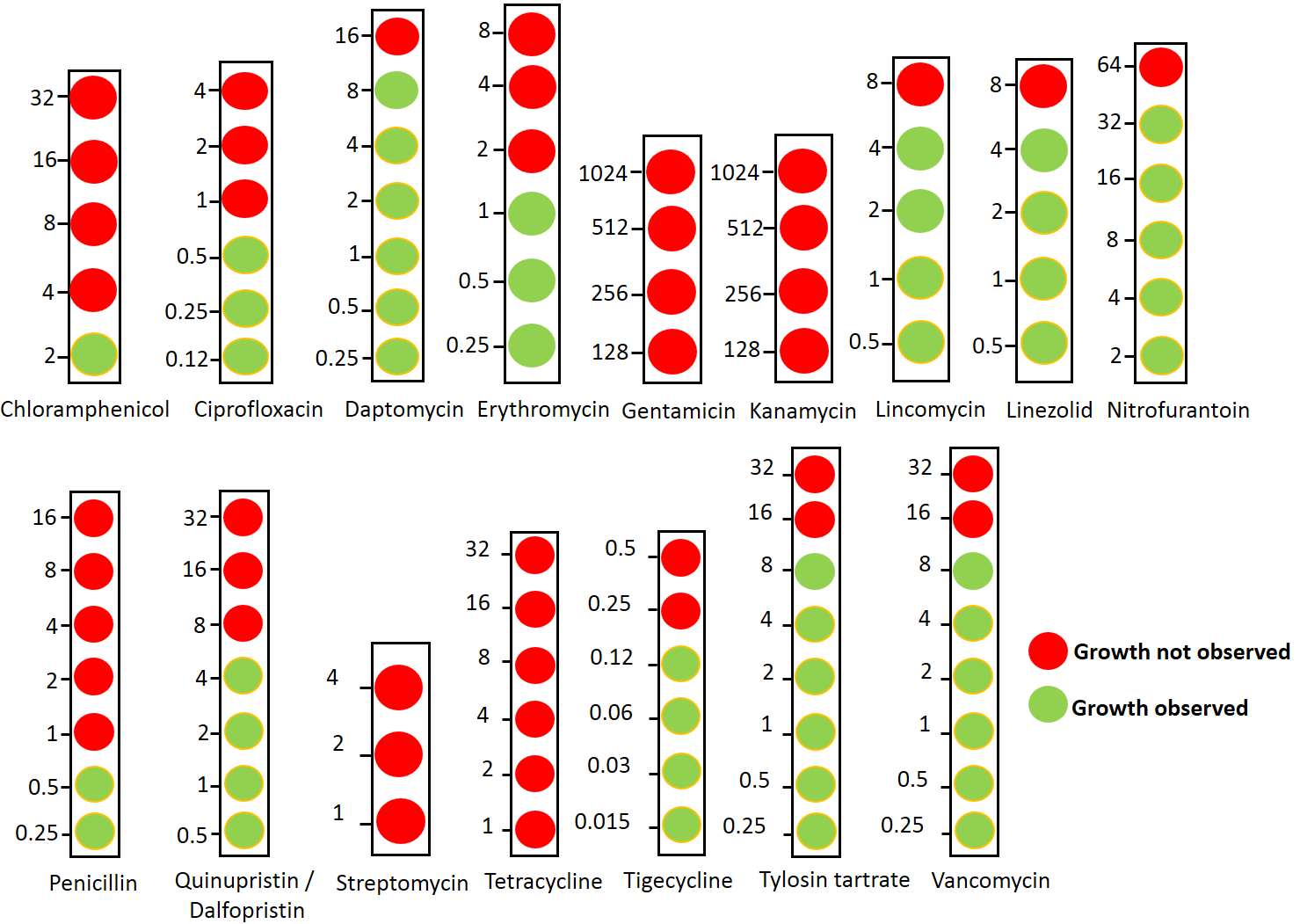


**Supplementary figure 4**


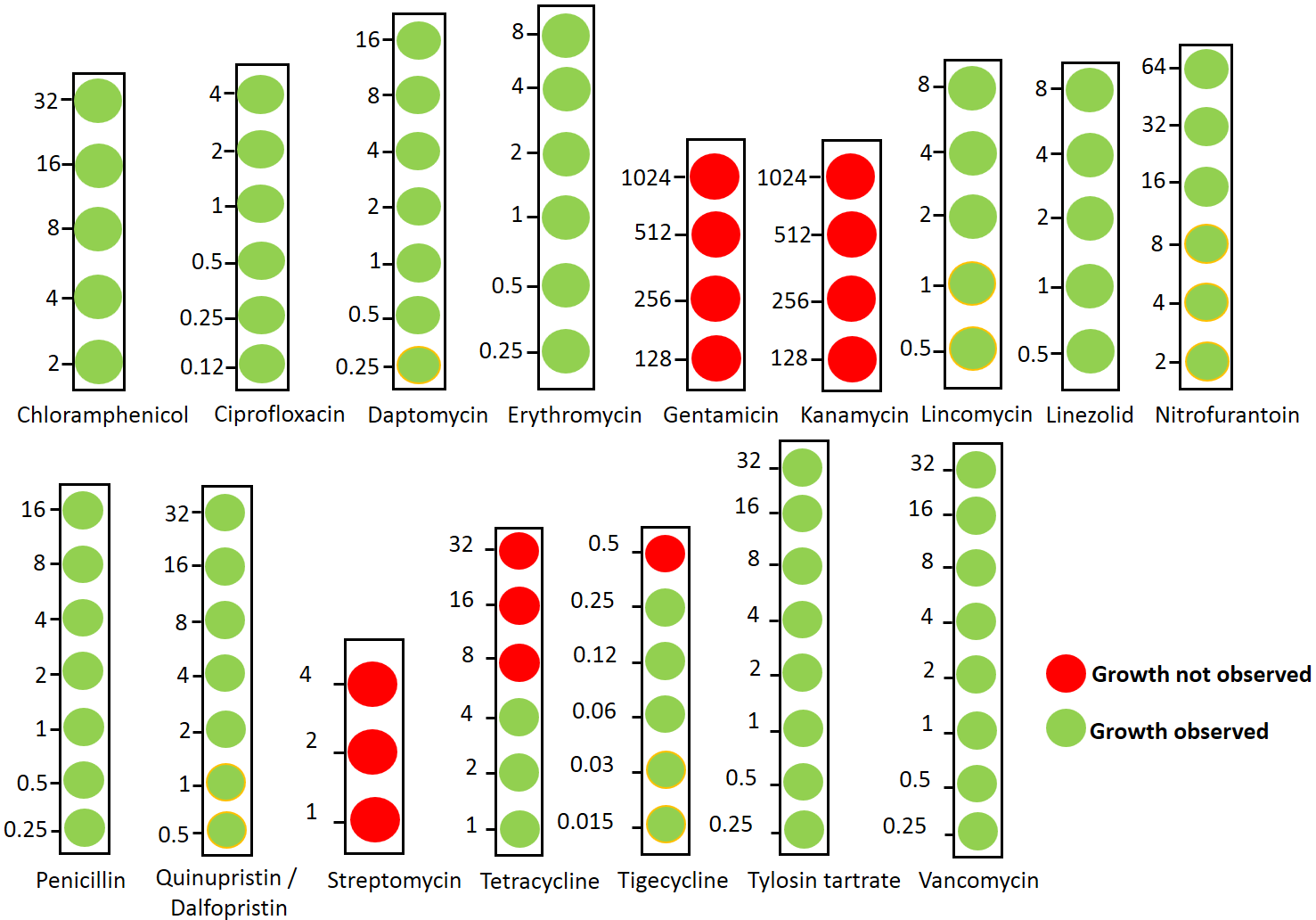

Supplement: Supplementary file 1 [file Data_Sheet_1.docx]
